# MIRELLA: a mathematical model explains the effect of microRNA-mediated synthetic genes regulation on intracellular resource allocation

**DOI:** 10.1093/nar/gkad151

**Published:** 2023-03-13

**Authors:** Federica Cella, Giansimone Perrino, Fabiana Tedeschi, Gabriella Viero, Carla Bosia, Guy-Bart Stan, Velia Siciliano

**Affiliations:** Istituto Italiano di Tecnologia-IIT, Largo Barsanti e Matteucci, Naples, Italy; Department of Bioengineering and Centre of Excellence in Synthetic Biology, Imperial College London, London, UK; Istituto Italiano di Tecnologia-IIT, Largo Barsanti e Matteucci, Naples, Italy; University of Naples Federico II, Naples, Italy; Institute of Biophysics, CNR Trento, Povo, Italy; Department of Applied Science and Technology, Politecnico di Torino, Torino, Italy; Italian Institute for Genomic Medicine, c/o IRCCS, Candiolo, Italy; Department of Bioengineering and Centre of Excellence in Synthetic Biology, Imperial College London, London, UK; Istituto Italiano di Tecnologia-IIT, Largo Barsanti e Matteucci, Naples, Italy

## Abstract

Competition for intracellular resources, also known as gene expression burden, induces coupling between independently co-expressed genes, a detrimental effect on predictability and reliability of gene circuits in mammalian cells. We recently showed that microRNA (miRNA)-mediated target downregulation correlates with the upregulation of a co-expressed gene, and by exploiting miRNAs-based incoherent-feed-forward loops (iFFLs) we stabilise a gene of interest against burden. Considering these findings, we speculate that miRNA-mediated gene downregulation causes cellular resource redistribution. Despite the extensive use of miRNA in synthetic circuits regulation, this indirect effect was never reported before. Here we developed a synthetic genetic system that embeds miRNA regulation, and a mathematical model, MIRELLA, to unravel the miRNA (MI) RolE on intracellular resource aLLocAtion. We report that the link between miRNA-gene downregulation and independent genes upregulation is a result of the concerted action of ribosome redistribution and ‘queueing-effect’ on the RNA degradation pathway. Taken together, our results provide for the first time insights into the hidden regulatory interaction of miRNA-based synthetic networks, potentially relevant also in endogenous gene regulation. Our observations allow to define rules for complexity- and context-aware design of genetic circuits, in which transgenes co-expression can be modulated by tuning resource availability via number and location of miRNA target sites.

## Graphical Abstract

**Graphical Abstract:**
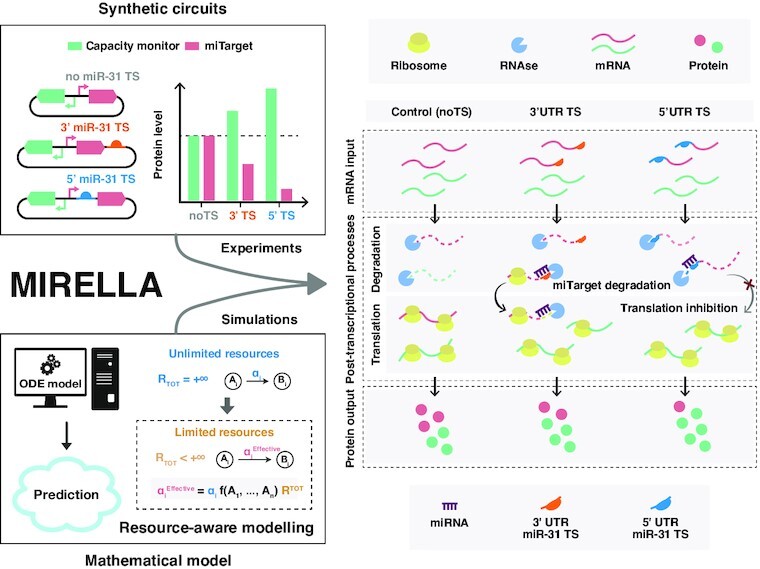
MIRELLA is a resource-aware mathematical model that by combining experimental data and simulations unveils the molecular mechanisms underlying the reallocation of cellular resources as a consequence of miRNAs activity.

## INTRODUCTION

Engineering mammalian cells with synthetic regulatory networks to obtain novel functionalities with predictable behaviour, requires a deep understanding of the dynamic interactions between the genetic circuits and the intracellular context in which they are intended to operate.

**Figure 1.**
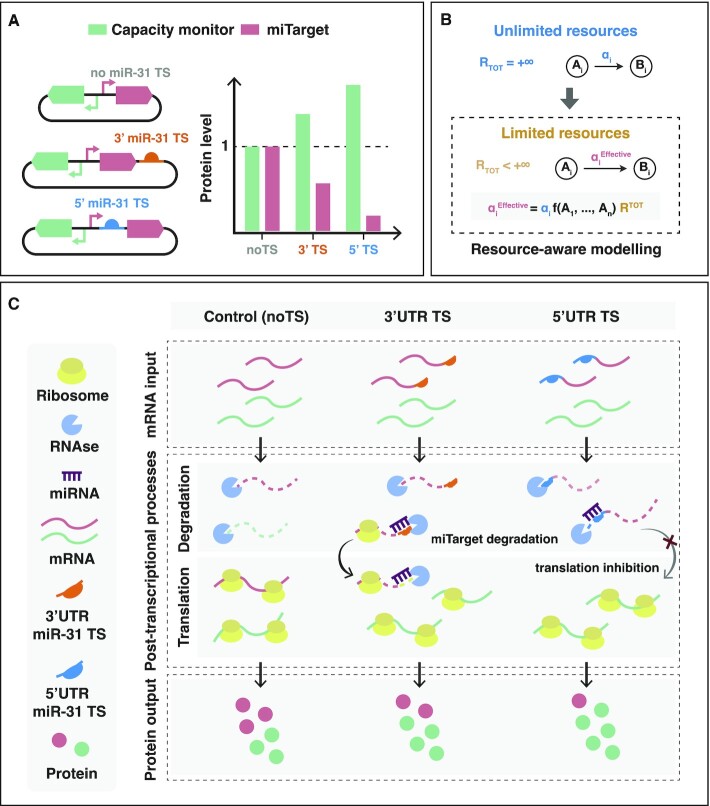
Graphical abstract of the study. (**A**) Graphical representation of the effect of the downregulation of a transgene (mKate-*miTarget*, red) by endogenous miRNAs (e.g. miR-31), on a co-expressed gene (*capacity monitor*, green) (1). The stronger the downregulation of the *miTarget*, the more the upregulation of the *capacity monitor*, in a miRNA target sites (TS) number- and location-fashion 5’UTR (blue) or 3’UTR (orange). (**B**) Modelling of gene networks in a resource-limited context. MIRELLA replaces all reaction rates (${\alpha }_i$) that involve shared cellular resources with their corresponding effective reaction rates (namely $\alpha _i^{Eff}$) that capture the availability of that resources according to the overall demand from competing genes (modelled via the general function $f( \ldots )$). (**C**) Effect of miRNA activity on protein expression in a finite-resource context. Control (noTS): protein expression in the absence of miRNA regulation. TS in the 5’ or 3’UTR: the slicer activity of miRNA-RISC complex triggers mRNA degradation of the *miTarget* (red) causing a queueing effect on the degradation of other mRNAs that results in *capacity monitor* (green) protein accumulation. TS in the 5’UTR: in addition to slicer activity, miRNA binding inhibits translation initiation, freeing up translational resources to the benefit of other transcripts (e.g. the *capacity monitor*). The resulting effect is stronger downregulation of *miTarget* and higher *capacity monitor* levels when TS are placed in the 5’UTR of the *miTarget*.

We recently showed that when exogenous DNAs are transiently delivered to mammalian cells, they compete for limited shared transcriptional and translational resources, reshaping RNA and protein levels, and leading to the coupling of otherwise independent genes (1,2). This becomes a pervasive problem, either when implementing regulatory circuits, or in attempts to carry out studies using system perturbations (i.e. overexpression or downregulation of a transient gene). The observation that synthetic circuits impose a gene expression burden to their host cells (1,3,4), prompted the development of ‘*context-aware*’ gene networks in which incoherent feedforward loops (iFFLs) that use biomolecular controllers such as endoribonucleases (2), or endogenous and synthetic miRNAs (1) were successfully exploited as burden mitigators. In miRNA-based iFFL, we observed that the protein levels of two independently expressed genes, one of which was regulated by miRNA, were strongly linked to the miRNA activity (1). As a point in case, using two co-expressed fluorescent proteins, EGFP and mKate, with mKate levels linked by design to the endogenous miRNA-31 (miR-31), we showed that the higher the number of miRNA target sites (TS) in the mKate UTRs, the stronger its downregulation, and the higher the levels of EGFP (Figure 1A). Conversely, by inhibiting miR-31, mKate levels increased, counterbalanced by reduced EGFP levels (1), supporting the miRNA-dependency of the observed effect. Results were robust to changing plasmid design (co-transfection *vs* single plasmid) and cellular context (1).

Given the biological importance and high applicability of miRNAs to synthetic circuits, we sought to investigate the molecular mechanisms involved in miRNA-dependent resource distribution. We postulate that this understanding will enable more precise circuits’ design with enhanced robustness and predictability, and may shed light on the secondary regulatory effect in endogenous pathways.

miRNAs are small non-coding RNAs produced from transcripts with stem-loop structures which undergo processing both in the nucleus and cytoplasm to be converted into mature, 21–26 nucleotides-long miRNAs. Mature miRNAs are assembled into the RNA-induced silencing complex (RISC) and bind their target mRNAs by base pairing usually to their 3’UTR or 5’UTR (5,6). Upon binding, miRNAs modulate their target through mRNA degradation and/or translational repression (7,8). Target sites (TS) can be fully or partially complementary to the miRNA. In the former case, the target is degraded through endonucleolytic cleavage (9), while in the latter translational repression dominates and transcript degradation occurs after deadenylation (7,8). Typically, in mammalian cells miRNAs bind to the 3’UTR of the endogenous target mRNA, and have non-perfect complementarity to the TS (7). Thus, their main mechanism of action is to repress translation. Moreover, miRNAs are often found in endogenous feedforward or negative feedback loops exploiting additional functions such as buffering gene expression against noise or fluctuations in external inducer concentration (10–13).

In synthetic biology, miRNAs have been repurposed as a versatile tool to build cell-specific devices, and have been largely used to create cell classifiers with biotechnological or biomedical applications (14,15) or to modulate the expression of the genetic devices (16,17). In these applications, to achieve strong downmodulation of the target genes, and increase the sensitivity to small concentrations of miRNAs, perfectly complementary target sites are typically used (18).

Here, we use a two-gene reporter system (EGFP hereafter named *capacity monitor*, and miR31TS-mKate, hereafter named *miTarget*), along with a mathematical model (MIRELLA) that qualitatively captures post-transcriptional events, to explain the effect of miRNA (MI) REgulation on intracellular resource aLLocAtion, effectively identifying key processes responsible for miRNA-based burden mitigation in mammalian cells (Figure 1A, B).

MIRELLA builds on an existing modelling framework (1) and considers that mRNA translation and degradation use pools of shared resources, among which we account as main players ribosomes and RNases, respectively. MIRELLA replaces reaction rates that involve shared cellular resources with effective reaction rates that account for the availability of each individual resource pool according to the overall gene expression demand (Figure 1B). We use MIRELLA to predict the effect of miRNA regulation on resource availability considering the strength of the downregulation (number of target sites), and the effect of TS location (i.e. as part of the 5’UTR or 3’UTR). The model suggests that miRNA-mediated downregulation of the target mRNAs causes a redistribution of translational resources (i.e. ribosomes) and impacts the RNA degradation machinery, overall contributing to a change in protein expression levels (Figure 1C). We then experimentally validated the model predictions for the expression of exogenous and endogenous genes.

Supported by the synergistic use of a mathematical model and experiments, our findings contribute to a deeper understanding of the mechanisms of miRNA operations in synthetic networks, which enables the resource-aware design of genetic circuits. Moreover, our results provide insights into secondary effects of miRNA regulation that might be potentially relevant also in endogenous gene regulation.

## MATERIALS AND METHODS

### DNA cloning and plasmid construction

Plasmid vectors carrying gene cassettes were created using In-Fusion HD cloning kit (Clontech), or digestion and ligation. Reaction included 1:2 molar ratio of plasmid backbone:gene inserts starting with 100 ng of vector backbone digested with selected restriction enzymes. All plasmids used in this study consist of a constitutive promoter driving the gene of interest. All plasmids used in this study were confirmed by sequencing analysis and are listed in Supplementary Table 2.

### Cell culture

H1299 cells were maintained in Roswell Park Memorial Institute medium (RPMI, Gibco) supplemented with 10% FBS (Atlanta BIO), 1% penicillin/streptomycin/l-glutamine (Sigma-Aldrich) and 1% non-essential amino acids (HyClone). The cells were maintained at 37°C and 5% CO_2_.

### Transfection

Transfections were carried out in 24-well plate format for flow cytometry analysis, in 12-well plate format for flow cytometry and qPCR analyses run on the same biological replicate or in 10 cm dishes for polysome profiling. H1299 cells were transfected with Lipofectamine® 3000 (Thermo Fisher Scientific) according to the manufacturer's instructions and 300 ng of total DNA for each sample for a transfection in 24-well plates and scaled up for larger formats. Details on transfections are provided in Supplementary Table 1.

### Flow cytometry and data analysis

Cells were analysed using a BD FACSAria™ cell analyser (BD Biosciences) using 488 nm and 561 nm lasers. Cells transfected in 12-well plates were washed with DPBS, detached with 100 μl of trypsin–EDTA (0.25%) phenol red and resuspended in 600 μl of DPBS (Thermo Fisher Scientific). 200 μl of cell suspension were used for flow cytometry and 400 μl for RNA extraction. For each analysis, 10 000+ events from each sample were recorded and data were normalised with three compensation controls: unstained (wild-type cells), and single colour controls (mKate only, EGFP only). Fluorescence intensity in arbitrary units (AU) was used as a measure of protein expression. Population of live cells was selected according to FCS/SSC parameters. Data analysis was performed with Cytoflow. For each sample, we gated the population of live cells and then the EGFP^+^mKate^+^ cells (Q2 quadrant in Supplementary Figure 9b). Within this population we calculated the geometric mean (Geo-Mean) of mKate and EGFP.

### Polysome profiling

Polysome profiling was performed following the protocols described in (19,20). To obtain the cytoplasmic lysates, cells were treated with cycloheximide (10 μg ml^−1^) for 3–4 min and then lysed in 300 μl of cold hypotonic lysis buffer (19). To remove nuclei, mitochondria and cellular debris, the lysates were centrifuged at 4°C for 5 min at 20 000 g. To separate ribosomal subunits, ribosomes and polysomes from other cytoplasmic molecules, the supernatant was loaded on a 10–40% (w/v) sucrose gradient and centrifuged for 1 h 30 min at 260 000 g at 4°C in a SW41 rotor using a Beckman Optima LE-80 Ultracentrifuge. Twelve 1 ml fractions were collected and the absorbance at 254 nm was monitored with the UA-6 UV/VIS detector (Teledyne Isco). RNA was purified fraction by fraction using the phenol/chloroform extraction method described in (21). The retro-transcription reaction was performed using the same volume of RNA for all polysomal fractions. The co-sedimentation profile of mRNAs was obtained by calculating the percentage (or fraction) of mRNAs in each fraction by qPCR as described in (22).

### mRNA half-life measurement upon DRB treatment

mRNA half-life was measured by treating H1299 cells 24 h post-transfection with 5,6-dichlorobenzimidazole 1-β-d-ribofuranoside–DRB (Sigma-Aldrich) 50 μM. Cells were collected at different time-points (0 h, 0 h 30 min, 1 h, 1 h 30 min, 2 h, 3 h, 4 h after treatment) for RNA extraction and qPCR analysis.

### mRNA extraction and reverse transcription

RNA extraction was performed with E.Z.N.A.® Total RNA Kit I (Omega Bio-tek). Protocol was followed according to the manufacturer's instructions and RNA was eluted in 30 μl of RNase-free water to maximise the yield. RNA samples were conserved at –80°C. The protocol was performed exclusively with RNase free water in an RNase-free environment.

PrimeScript RT Reagent Kit with gDNA Eraser—Perfect Real Time (Takara) was used according to manufacturer's instructions. The protocol was performed on ice in a RNase-free environment to avoid RNA degradation. A negative control without PrimeScript RT Enzyme Mix I was always prepared to be sure that samples were not contaminated with genomic DNA.

### qPCR

Fast SYBR Green Master Mix (Thermo Fisher Scientific) was used to perform qPCR of cDNAs obtained from 500 ng of RNA and diluted 1:5. Samples were loaded in MicroAmp™ Fast Optical 96-Well Reaction Plate (0.1 ml) and the experiment was carried out with a 7900HT™ Fast machine. Each well contained 10 μl of final volume (5 μl SYBR Green Master Mix 2X, 2 μl ddH2O, 1 μl of each primer, 1 μl of template). Also, a control without template (blank) was set. Primers were designed to amplify a region of 60–200 bp (Supplementary Table 3) and with a temperature of annealing between 50°C and 65°C. Data were analysed using the Comparative Ct Method according to Applied Biosystems Protocols.

### Modelling

We constructed a deterministic ODE model to qualitatively capture post-transcriptional events in order to identify key processes responsible for miRNA-based resource reallocation. Our ODE model is based on a previously published resource-aware modelling framework (1). Full details of its formulation and parameterisation can be found in Supplementary Notes 1–6, Supplementary Tables 4–7, and Supplementary Figures 1–2, 6–7.

### Deterministic simulations of the ODE model

All the simulations of the ODE model were run using Python 3 (v. 3.8.13). The *scipy.integrate.solve_ivp* function from the *SciPy* library (v. 1.8.0) was used to numerically integrate the ODE model. More specifically, we used the *Radau* method (stiff ODE solver) with an absolute tolerance of ${10}^{ - 9}$, and a relative tolerance of ${10}^{ - 6}$. To simulate the ODE model at steady state, the time span was set to [0, 10 000] h. Hence, steady state was taken as the value for each molecular species at the end of a numerical simulation of 10 000 h.

The parameter values used to simulate the ODE model were chosen as described in Supplementary Note 6 and reported in Supplementary Table 5 (for simulations of the translational resource reallocation) and Supplementary Table 7 (for simulations of the degradation resource reallocation). All plots were generated in Python using the *Matplotlib* library (v. 3.5.1). The code to run all the simulations and the model is available publicly as Jupyter notebooks (https://github.com/giansimone/MIRELLA/).

### Analytical characterisation of the translational resource reallocation

To quantitatively characterise the reallocation of the translational resources at steady state, we derived an analytical solution of the ODE model as reported in Supplementary Note 1. Full details of the analytical solution derivation can be found in Supplementary Note 4. The steady-state expression levels for both the *miTarget* (${\bar{p}}_T$) and the *capacity monitor* (${\bar{p}}_C$) are as follows:


(1a) $$\begin{equation*}{\bar{p}}_T = \frac{{{\gamma }_T}}{{{\delta }_T}} \cdot \frac{{{\rho }_T + \sigma \ \rho _T^Q}}{{1 + {\rho }_C + {\rho }_T + \sigma \ \rho _T^Q}} \cdot {r}^{Total}\end{equation*}$$

(1b) $$\begin{equation*}{\bar{p}}_C = \frac{{{\gamma }_C}}{{{\delta }_C}} \cdot \frac{{{\rho }_C}}{{1 + {\rho }_T + \sigma \ \rho _T^Q + {\rho }_C}} \cdot {r}^{Total}\end{equation*}$$

where ${\rho }_T$ is the resource demand coefficient for the *miTarget*, $\rho _T^Q$ is the resource demand coefficient for the *miTarget:miRNA* complex, and ${\rho }_C$ is the resource demand coefficient for the *capacity monitor*. The boolean parameter $\sigma \ \in \ \{ {0,\ 1} \}$ captures the location of the miR-TS at either the 5’ ($\sigma \ = \ 0$) or 3’ ($\sigma \ = \ 1$) UTRs. The analytical expressions of the resource demand coefficients are as follows:


(2a) $$\begin{equation*}{\rho }_T = \frac{{{n}_T\ {\alpha }_T}}{{{\kappa }_T\ \left( {{\beta }_T + \frac{{{\alpha }_Q\ {\eta }^ + \ \beta _T^Q}}{{{\beta }_Q\ \left( {\beta _T^Q + {\eta }^ - } \right)}}} \right)}}\end{equation*}$$

(2b) $$\begin{equation*}\rho _T^Q = \frac{{{n}_T\ {\alpha }_T}}{{{\kappa }_T\ \left( {\beta _T^Q + \frac{{{\beta }_T\ {\beta }_Q\ \ \left( {\beta _T^Q + {\eta }^ - } \right)}}{{{\alpha }_Q\ {\eta }^ + }}} \right)}}\end{equation*}$$

(2c) $$\begin{equation*}{\rho }_C = \frac{{{n}_C\ {\alpha }_C}}{{{\kappa }_C\ {\beta }_C}}\end{equation*}$$

where ${\kappa }_T$ and ${\kappa }_C$ are the effective dissociation constant for the *miTarget* and *capacity monitor*, respectively (see Supplementary Note 1 for their definitions). All other parameters are described in Supplementary Table 5.

### Model fitting

**Figure 2.**
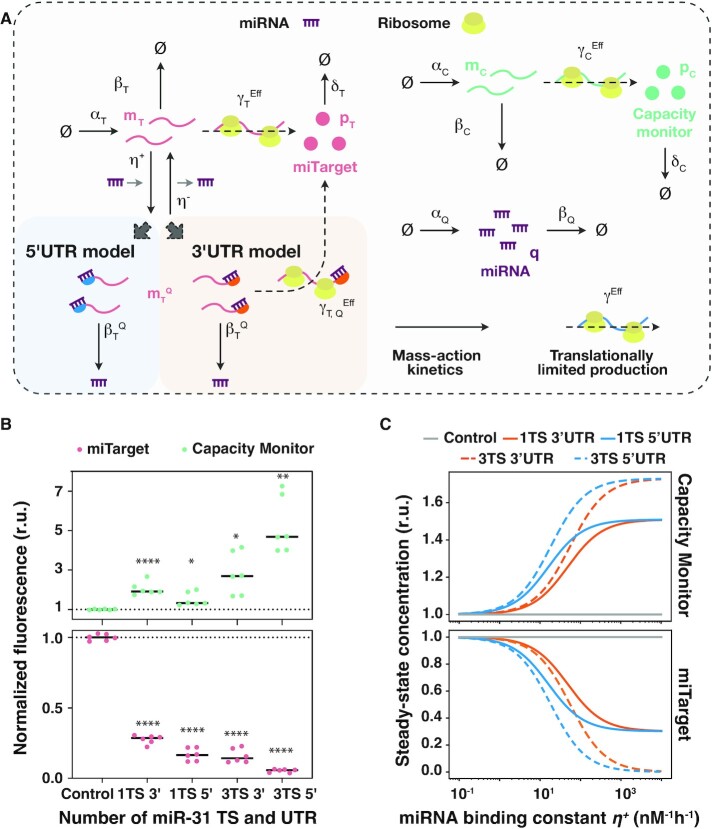
MIRELLA modelling framework used to capture miRNA-effect on resource allocation. (**A**) Graphical representation of the resource-aware model used to study the reallocation of the translational resources (ribosomes). For each of the two exogenous genes, the model captures the essential features of translation, degradation, and interactions between genes and ribosomes. Initially, the model does not capture the interactions between genes and RNases since the shared cellular resource pool for RNA degradation (RNases) is here considered unlimited. The competition for the shared pool of ribosomes is captured via the effective translation rate constants $\gamma _T^{Eff}$ and $\gamma _{T,\ Q}^{Eff}$ (*miTarget*), and $\gamma _C^{Eff}$ (*capacity monitor*). The miRNA mode of action changes according to the location of the miR-TS and generates two different models: the ‘3’ UTR model’ and the ‘5’ UTR model’. The strength of the miRNA regulation is modelled via the miRNA-to-*miTarget*-mRNA binding constant ${\eta }^ +$. (**B**) Fold change of miTarget and capacity monitor protein levels compared to control (noTS) set to 1. Flow cytometry data were acquired 48 h post-transfection and plotted as mean. r.u.: relative units. *N* = 6 biological replicates. Unpaired two-sided *t*-test. *P* value: ****<0.0001, **<0.005, *<0.05. Dataset taken from (1). (**C**) Predicted steady-state protein levels of the *miTarget* (bottom) and the *capacity monitor* (top) varying the miRNA binding constant ${\eta }^ +$ when considering different design conditions. Each colour represents a different design condition that depends on the location and the number of the miRNA target sites within the UTRs of the *miTarget* gene, i.e. 1 or 3 TS, either in the 3’ or the 5’ UTR. The miRNA binding constant ${\eta }^ +$ is considered as an independent variable and thus is not set to a fixed value. The values considered for ${\eta }^ +$ span a range of reasonable characteristic values. r.u.: relative units. A description of the model can be found in Supplementary Notes 1–3. All the molecular species captured in the model are listed in Supplementary Table 4, whilst all the model parameters—including the numerical values used for the presented simulation results—are summarised in Supplementary Table 5.

The model fitting shown in Supplementary Figure 3 was performed using Python 3 (v. 3.8.13). The *scipy.optimize.differential_evolution* function from the *SciPy library* (v. 1.8.0) was used to find the model parameters that best fit the predicted steady-state expression levels for both the *miTarget* and *capacity monitor* to the experimental data reported in Figure 2B. The error distance between the predicted and the experimental data was evaluated using the following loss function:


$$\begin{equation*}L\left( \vartheta \right) = {\left| {\left| {y - \hat{y}\ } \right|} \right|}_2^2 + \lambda \ {\left| {\left| {\ \vartheta \ } \right|} \right|}_2^2\end{equation*}$$

where $y\ = \ {[{y}_{Control},\ {y}_{1TS\ 3^{\prime}},\ {y}_{1TS\ 5^{\prime}},\ {y}_{3TS\ 3^{\prime}},\ {y}_{3TS\ 5^{\prime}}]}^T$ is the vector that contains the different mean values for the *miTarget* (mKate) and the *capacity monitor* (EGFP) in each condition as reported in Figure 2b, $\hat{y} = {[{\hat{y}}_{Control},\ {\hat{y}}_{1TS\ 3^{\prime}},\ {\hat{y}}_{1TS\ 5^{\prime}},\ {\hat{y}}_{3TS\ 3^{\prime}},\ {\hat{y}}_{3TS\ 5^{\prime}}]}^T$ is the vector that contains the predicted values in the different simulated conditions, and $\vartheta$ is the vector that contains the model parameters that have to be identified via the model fitting. An ${L}_2$-regularisation term was added to the loss function to prevent an ill-conditioned parameter estimation problem. To keep all the model parameters within the same order of magnitude, the regularisation hyperparameter was set to $\lambda \ = \ 0.001$.

The plot in Supplementary Fig. 3 was generated in Python using the *Matplotlib* library (v. 3.5.1). The code to perform the model fitting is available publicly as a Jupyter notebook (https://github.com/giansimone/MIRELLA/).

## RESULTS

A modelling framework explains the relation between gene downregulation by microRNAs and cellular resources reallocation

We previously observed that miRNA-based iFFL circuit designs can be used to reduce the resource-based coupling of two co-expressed genes, and that miRNA-driven gene downregulation is associated with increased expression of co-encoded, independent genes (1). To understand the mechanisms underlying this indirect effect on independent genes expression, we developed MIRELLA, a deterministic resource-aware model that captures the resource-constrained co-expression of two constitutive genes when one of them is downregulated by an endogenous miRNA (Figure 2A). With a focus on miRNA activity as a key player in iFFL-based burden mitigation, MIRELLA extends existing models of biochemical reactions by taking into account the effects of shared cellular resources. This is achieved through the use of effective reaction rates that depend on cellular resources demand, like previously done in (1), while explicitly accounting for ribosomes and RNases resource pools (Figure 1B). In what follows, we provide a brief overview of the model, whilst its full details can be found in Supplementary Note 1.

**Figure 3.**
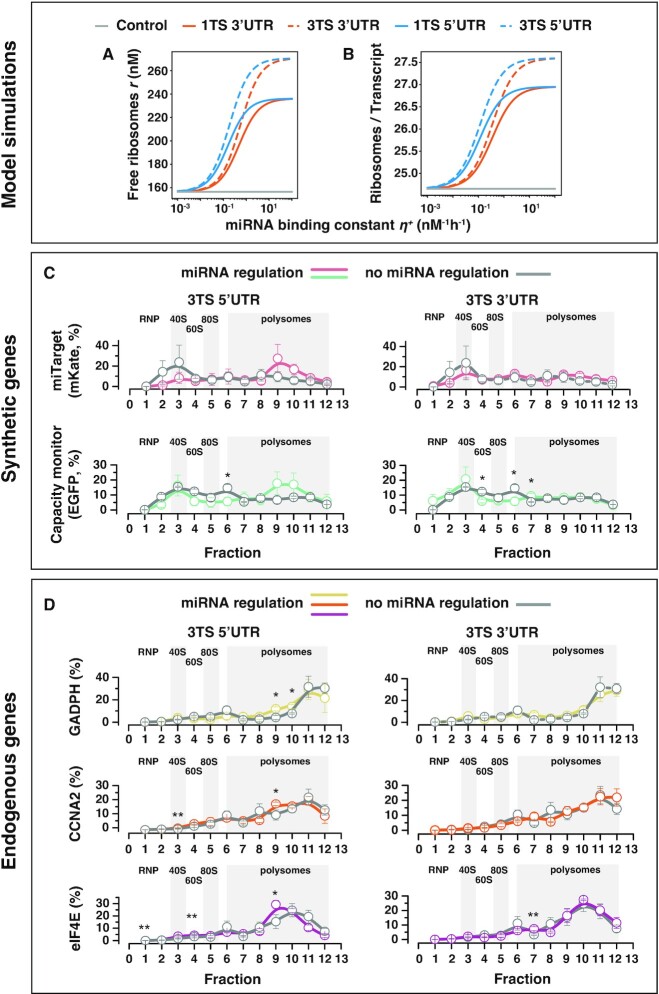
Ribosomes redistribution. (**A**) Predicted steady-state concentration levels of the free ribosomes varying the miRNA binding constant ${\eta }^{+}$ in different design conditions. (**B**) Predicted steady-state ribosomal densities (Materials and Methods) for both the *miTarget* and *capacity monitor* genes varying the miRNA binding constant ${\eta }^{+}$ in different design conditions. Design conditions include different location and number of the miRNA target sites within the UTRs of the *miTarget* gene. The miRNA binding constant ${\eta }^ +$ is considered as an independent variable and thus is not set to a fixed value. Instead, the values considered for ${\eta }^ +$ span a range of reasonable characteristic values. An increase in the miRNA binding constant ${\eta }^ +$ is correlated to an increase in the pool of free ribosomes and the ribosomal densities, and supports the hypothesis of ribosomes reallocation upon miRNA-driven regulation. (C, D) Relative distribution of transcripts and co-sedimentation analysis in polysomes profiles in H1299 cells transfected with a bidirectional promoter plasmid encoding for EGFP (*capacity monitor*) and mKate (*miTarget*) with miR-31 TS either at the 5’ or 3’ UTR. As control, we used the same plasmid lacking the miR-31 target sites. We analysed both synthetic (**C**) and endogenous (**D**) genes. Polysome profiles were obtained 48 hours post transfection. Means of the relative percentage of transcript sedimentation along the profile ± SE. SE: standard errors are shown. *N* = 3 biological replicates. Unpaired two-sided *t*-test. *P*-value: * < 0.05, ** < 0.005.

**Figure 4.**
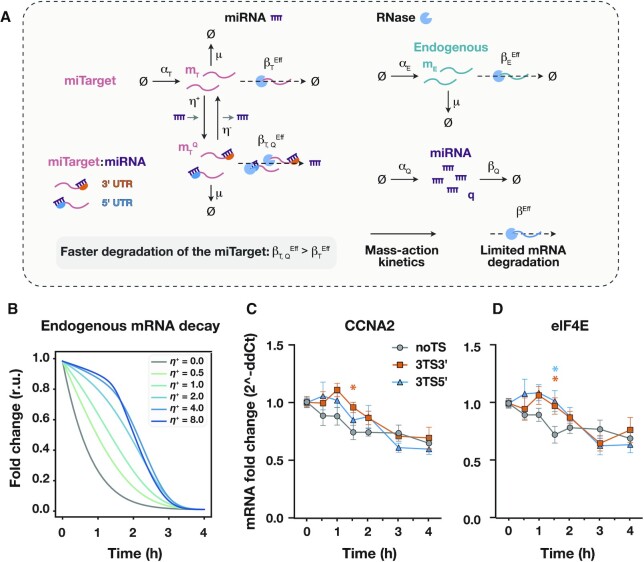
miRNA action increases non-target transcripts' half-life. (**A**) Graphical representation of the resource-aware model used to study the reallocation of the degradation resources (RNases). The model described in Figure 2 is extended to include a finite pool of RNases. The competition for the shared pool of RNases is captured via the effective mRNA degradation rate constants $\beta _T^{Eff}$ and $\beta _{T,\ Q}^ +$ (*miTarget*), and $\beta _E^{Eff}$ (*endogenous* gene). The miRNA-driven decay increases the rate of binding between the *miTarget* transcript and the degrading resources by increasing the association constant $\beta _T^ +$ to the new value $\beta _{T,\ Q}^ +$. A description of the model can be found in Supplementary Note 5. All the molecular species captured in the model are listed in Supplementary Table 6, whilst all the model parameters—including the numerical values used for the simulations—are summarised in Supplementary Table 7. (**B**) Predicted mRNA degradation profiles of an endogenous gene upon halting of transcription for different values of the miRNA binding constant ${\eta }^ +$. Reallocation of degrading resources alters the endogenous mRNA degradation profile and depends on the miRNA binding constant. The mRNA degradation profiles were normalised to the initial data point. Each colour represents a different value for the miRNA binding constant ${\eta }^ +$. r.u.: relative units. **(c, d)** mRNA half-life and degradation dynamics measurement of CCNA2 (**C**) and eIF4E (**D**) upon DRB treatment (50 μM). All data were acquired 24 hours post transfection and are plotted as mean ± SE. SE: standard error. *N* = 4–8 biological replicates. An unpaired two-sided *t*-test was used to compare 3TS3’ and 3TS5’ samples to the noTS control. *P*-value: * < 0.05.

Since miRNAs act post-transcriptionally, the model focuses on the processes that contribute to proteins’ expression, namely miRNA-dependent mRNA downregulation, mRNA degradation and mRNA translation. The driving hypothesis is that the observed increased expression of an independent gene following the downregulation of the miRNA target is likely due to the reallocation of shared cellular resources involved in post-transcriptional processes. The model thus consists of a set of ordinary differential equations (ODEs) that capture the relative gene expression levels of a constitutive gene which is modulated by an endogenous miRNA (*miTarget*—Figure 2A), and a second constitutive gene, whose expression levels reflect variations in the availability of shared cellular resources (*capacity monitor*—Figure 2A). The *miTarget* is a mRNA encoding for a red fluorescent protein (mKate) that includes target sites for miR-31 in its 5’ or 3’ UTR, whereas the *capacity monitor* is a constitutively expressed green fluorescent reporter (EGFP). For each of these two genes, the model captures the essential features of transcription, translation, degradation, and also the interactions between genes and shared cellular resource pools as illustrated in Figure 2A. The model assumes that the shared cellular resource pool for translation (here comprising only ribosomes for simplicity) is constant, whereas the pool for RNA degradation (e.g. RNases) is initially considered unlimited. This enables us to initially neglect the demand for RNA degradation resources and to focus on how miRNA-driven regulation impacts ribosomes reallocation (Figure 2A and 3A, B). In a second step, we consider a finite pool of RNases, which are reallocated depending on degradation demand from the co-expressed genes (Figure 4A, B). Focusing on post-transcriptional events, the model does not explicitly consider shared transcriptional resource pools. This choice does not affect the results and the conclusions since variations in transcriptional burden can be accounted by a change in the transcription rate constants ${\alpha }_T$, ${\alpha }_C$, and ${\alpha }_Q$ as shown in Supplementary Note 2. The core elements of the model include the effective translation rate constants for both the *miTarget* ($\gamma _T^{Eff}$) and *capacity monitor* ($\gamma _C^{Eff}$) genes (Supplementary Note 1). These rates change dynamically according to the translational resource demand, which depends on the mRNA expression levels ${m}_T$ and ${m}_C$, and on the effective dissociation constants ${\kappa }_T$ and ${\kappa }_C$ (see Supplementary Note 1 for further details). The model considers two main modes of action of miRNAs on their target genes, namely regulation of translation initiation (23,24) and mRNA degradation (25). Since synthetic circuits commonly use perfectly complementary TS to maximise the downregulation, we consider mRNA degradation the main outcome of miRNAs activity. However, when located in proximity of the AUG at the 5’ UTR, we additionally consider a steric hindrance effect of miRISC complex that competes with ribosome binding and impairs mRNA translation (26) (Figure 1C).

To capture the binding interactions between the *miTarget* mRNA and its cognate miRNA, the model considers a further molecular binding reaction as illustrated in Figure 2A. The strength of the miRNA regulation is modelled via the characteristic parameter associated with the binding reaction between the *miTarget* mRNA and its cognate miRNA, that is the miRNA binding constant ${\eta }^ +$ (Figure 2A). ${\eta }^ +$ can be used as a proxy for capturing the effect of different repetitions of TS in the 5’ or 3’ UTRs of the target mRNA. Hereafter, the binding constant ${\eta }^ +$ is assumed to be proportional to the number of TS (i.e. we do not consider cooperation effects in miRNA-driven regulation). The ‘3’ UTR model’ (shaded orange region in Figure 2aA) considers miRNA regulation via the mRNA degradation rate $\beta _T^Q$, whilst the ‘5’ UTR model’ (shaded blue region in Figure 2A) considers miRNA regulation via both the mRNA degradation rate $\beta _T^Q$ and the effective translation rate $\gamma _{T,\ Q}^{Eff}$ (Supplementary Note 1). We assume first-order miRNA-mediated degradation of the target mRNA *miTarget*, which results in an increase of the mRNA degradation rate by a factor $\lambda _T^Q$ > 1, that is $\beta _T^Q$ = $\lambda _T^Q \times {\beta }_T$. When the TS are within the 5’ UTR, the model assumes that the effective translation rate $\gamma _{T,\ Q}^{Eff}$ is equal to zero since, in this case, *miTarget:miRNA* transcripts cannot be translated due to steric hindrance.

To check if our model correctly predicts protein expression upon miRNA regulation, we ran simulations of the resource-aware model and compared the results with the experimental data published in (1). Data represent the protein levels of *miTarget* and *capacity monitor* in H1299 cells, which naturally express high levels of miR-31. *miTarget* and *capacity monitor* are under the regulation of a bidirectional constitutive CMV promoter (Supplementary Figure 9a). The *miTarget* includes either 1 or 3 TS in the 3’ or in the 5’ UTR. The higher the number of TS, the stronger the repression exerted by miR-31, and consequently, the higher the *capacity monitor* levels (Figure 2B).

We simulated the presence of multiple miRNA TS in both the 3’ UTR and 5’ UTR by considering a range of reasonable characteristic values for the miRNA binding constant ${\eta }^ +$ (Supplementary Table 5 and Supplementary Note 6), and monitored at the steady-state the values of the molecular species of the system (Materials and Methods). Simulation results confirmed that the resource-aware model can recapitulate the steady-state protein levels of the *miTarget* and the *capacity monitor* observed in the experimental data (Figure 2C). We found that miRNA binding constant ${\eta }^ +$ is positively correlated with the steady-state protein levels of the *capacity monitor*. As expected, the simulation results showed that when miRNA TS are located in the 5’ UTR there is stronger downregulation of the *miTarget* followed by higher *capacity monitor* levels (Figure 2C).

We next solved the model analytically to obtain insights into resource demands that account for the steady-state levels of both the *miTarget* and the *capacity monitor* proteins (Eq. (1), Materials and Methods; full details in Supplementary Note 4). We found that these levels depend on the resource demand coefficients ${\rho }_T$ (for *miTarget* translation, Eq. (2a)), ${\rho }_C$ (for *capacity monitor* translation, Eq. (2c)), and $\rho _T^Q$ (for *miTarget:miRNA* translation, Eq. (2b)). We noticed that the resource demand coefficient $\rho _T^Q$ is non-zero only when TS are present in the 3’UTR (Eq. (2b)). We also observed that the miRNA regulation directly alters the resource demand coefficients associated with *miTarget* translation, although in different ways. More specifically, the coefficient ${\rho }_T$ (Eq. (2a)) is downregulated by both the miRNA binding constant ${\eta }^ +$ and the miRNA-enhanced degradation rate $\beta _T^Q$. In contrast, the demand coefficient $\rho _T^Q$ (Eq. (2b)) is upregulated by the miRNA binding constant ${\eta }^ +$ and downregulated by the miRNA-enhanced degradation rate $\beta _T^Q$. Therefore, the miRNA-driven *miTarget* degradation reduces the resource demand coefficients, and hence the amount of *miTarget* mRNA that is translated into *miTarget* proteins. This effect leads to a redistribution of the translational resources on the *capacity monitor* transcripts, which in turn increases the amount of *capacity monitor* proteins. This is the only source of translational resource redistribution in the 3’ UTR model, whereas the absence of the coefficient $\rho _T^Q$ (i.e. $\rho _T^Q$ = 0) when TS are present in the 5’ UTR amplifies the redistribution effect in line with the experimental data shown in Figure 2B. We further validated the resource-aware model by fitting the model parameters to the experimental data as shown in Supplementary Figure 3 (Materials and Methods).

### Regulation by miRNA binding to the 5’UTR results in ribosomes reallocation and modified translational profiles

To explore the effect of miRNA-driven reallocation of translational resources we first used our resource-aware model to predict the amount of free and translating ribosomes at steady state. Based on the results of the simulation reported in Figure 2C, the pool of free ribosomes positively correlates with the miRNA binding constant ${\eta }^ +$ (Figure 3A), and with the location of miRNA-TS. Furthermore, the *ribosomal density* defined here as the number of translating ribosomes *per* total number of transcripts, positively correlates with the miRNA binding constant ${\eta }^ +$ for both the *miTarget* and the *capacity monitor* (Figure 3B). This suggests that the miRNA-driven downregulation of the *miTarget* frees up translational resources, which can be deployed to the translation of other transcripts, including co-expressed ones such as the *capacity monitor*. We investigated what may cause the ribosomes reallocation by analytically solving the model at the steady state (Supplementary Note 4). We derived the steady-state level for the pool of free ribosomes ($\bar{r}$) and found that it is inversely proportional to the sum of the resource demand coefficients, that is $\bar{r}\ \sim\ \frac{1}{{{\rho }_T + \rho _T^Q + {\rho }_C}}$ (Supplementary Note 4). Since miRNA regulation does not affect the *capacity monitor* resource demand coefficient, a reduction in the total *miTarget* resource demand (i.e. ${\rho }_T$ + $\rho _T^Q$) corresponds to an increase in the amount of free ribosomes (Supplementary Note 4). We then analytically calculated the steady-state ribosomal densities for the *capacity monitor* transcripts and found that these correlate with the amount of free ribosomes (Supplementary Note 4). Of note, our simulations and the analytical solution show an increased number of ribosomes per transcript also for the *miTarget* mRNA when the miR-TS are located at the 5’ UTR (Figure 3B, Supplementary Note 4). Even if counterintuitive, this suggests that the increased amount of available ribosomes is to the benefit of *miTarget* transcripts that did not undergo miRNA-mediated downregulation.

Next, we experimentally validated the model predictions. We performed polysome profiling in H1299 cells transfected with *miTarget* and *capacity monitor* to observe the changes in co-sedimentation of the reporter mRNAs with polysomes (19,20) following miRNA modulation. We considered several conditions, including no miRNA regulation and TS inserted in the 5’ or 3’ UTR (Figure 3C, D). After sucrose gradient separation of cytoplasmic lysates, it is possible to follow the sedimentation of transcripts with free cytosolic light components (ribonucleoproteins, RNPs), ribosomal subunits (40S and 60S) and monosomes (80S) - all associated with non-translating particles - and with polysomes comprising translating transcripts bound by multiple ribosomes (Figure 3C, D). In line with model predictions, both the *capacity monitor* (EGFP, Figure 3C, bottom) and the *miTarget* (mKate, Figure 3C, top) exhibit modified co-sedimentation profiles upon miR-31 modulation as compared to the control (no miRNA regulation, grey line). In addition, we monitored the co-sedimentation profiles of endogenous transcripts, i.e. eIF4E, CCNA2 and GAPDH, that we previously observed to be impacted by resource competition. Following miRNA regulation of *miTarget*, we found that they also benefit from ribosome reallocation (Figure 3D). Our data indicate that for all analysed genes, there is a shift towards the polysomal fractions (peaks 9–10) that is more pronounced when the miR-31 TS are placed at the 5’ UTR (Figure 3C, D, left) as compared to the 3’ UTR (Figure 3C, D, right) of the *miTarget*. These results are consistent with our hypothesis stating that, due to the physical proximity of the TS to the AUG, the miRISC complex interferes with the binding of ribosomes to the target mRNA, resulting in their re-allocation on other transcripts.

### microRNA activity induces a queuing effect on mRNA degradation

Along with ribosome redistribution, we used MIRELLA to investigate the impact of miRNA-driven regulation on the RNA degradation machinery. To this end, we modified our model to include a finite pool of RNases (Supplementary Note 5), recapitulating the essential features of transcription, degradation, and interactions between transcripts and the degrading resource pool (RNases) as illustrated in Figure 4A. We then simulated the degradation of an *endogenous* transcript upon halting of transcription (i.e. all transcription rate constants were set to zero after the system reached its steady state) for different reasonable values of the miRNA binding constant ${\eta }^ +$ (Supplementary Table 7 and Supplementary Note 6).

The competition for the shared pool of RNases is captured via the effective mRNA degradation rates derived for the *miTarget* ($\beta _T^{Eff}$) and the *endogenous* ($\beta _E^{Eff}$) gene (Figure 4A and Supplementary Note 5). The effective degradation rates approximate the degradation reactions shown in Supplementary Fig. 2 with simpler first-order decay reactions (Figure 4A and Supplementary Note 5). These rates change dynamically and depend both on the expression levels of the degrading *mRNA:RNase* complexes (${s}_T$ and ${s}_E$, Supplementary Fig. 2) and the association and dissociation constants between the RNase and the mRNA strands (Supplementary Note 5 for further details). We assume that the miRNA-driven decay increases the rate of binding between the *miTarget* transcript and the molecular species of the degradation machinery by increasing the association constant $\beta _T^ +$ to the new value $\beta _{T,\ Q}^ +$ (Supplementary Fig. 2). This effect produces an increase in the effective mRNA degradation rate $\beta _T^{Eff}$ to the new value $\beta _{T,\ Q}^{Eff}$ (Figure 4a). The model predicted an altered degradation profile depending on the miRNA binding constant ${\eta }^ +$ (Figure 4B, extended data presented in Supplementary Fig. 4) and showed that the endogenous degradation dynamics are slower when compared to the control (${\eta }^ +$ = 0). Moreover, the endogenous degradation profile presents two different decay phases when the miRNA binding constant ${\eta }^ +$ is greater than ∼2 nM^−1^ h^−1^ (Figure 4B and Supplementary Figure 4b). Specifically, the endogenous mRNA levels remain almost stable during the first decay phase, whereas they degrade quicker during the second phase. We hypothesise that the increased degradation of the target by miR-31 (Supplementary Figure 4a) sequesters a significant portion of the degradation machinery (Supplementary Fig. 4c, d), which is thus not available for the degradation of non-target mRNAs (Figure 4A and Supplementary Figure 4b, e). We also observed that the decay dynamics of the non-targeted transcripts become faster once the majority of *miTarget* transcripts are degraded (Supplementary Figures 4 and 5), probably due to the reallocation of degrading resources from the *miTarget* mRNAs to the non-target mRNAs (Supplementary Figure 4d, e).

To experimentally validate the model predictions, we treated cells with 5,6-dichloro-1-beta-d-ribobenzimidazole (DRB), a transcription inhibitor (27) and measured the mRNA half-life of the endogenous genes CCNA2 and eIF4E, whose half-life are reported within the range 2–4 h (28) (Figure 4C, D). We selected these genes since their mRNA half-life is much shorter than that of the *capacity monitor* EGFP, which is roughly >8 h (29) and thus does not permit to capture faster decay dynamics.

We transfected H1299 cells with mKate encoding plasmid with noTS or 3 miR-31 TS at either the 3’ or the 5’UTR along with a constitutively expressed EGFP. Over a time-frame of 4 h, CCNA2 and eIF4E mRNAs exhibit a longer decay time when mKate is flanked by miR-31 TS as compared to the control (no miR-31 TS) (Figure 4C, D). More specifically, the two mRNA species decrease immediately after treatment only in the control, while in the case of 3TS at the 3’ and 5’ UTRs, they remain almost stable for 1 h 30 min after treatment, undergoing a significant decrease after 2–3 h. The qualitative trend of the 3TS 3’ and 3TS 5’ samples are very similar and in agreement with our hypothesis that miRNAs enhance target degradation at the same rate when they bind to 3’- or 5’-UTR TS. Finally, the experimental measurements show that the two mRNA species reach a plateau 4 h post-DRB treatment, in agreement with the model predictions. Although the predicted and experimental decays exhibit the same dynamics, we find that the endogenous mRNAs are not completely degraded upon treatment with DRB, which we argue is a limitation of the mRNA half-life assay. In fact, we measured CCNA2 and eIF4E mRNAs up to 6 h post-DRB treatment in wild-type cells and observed that it reaches about 50% of the initial concentration for both genes (Supplementary Figure 8). Overall, our results confirm the model predictions and are in agreement with our hypothesis of a prominent queuing effect on the mRNA degradation pathway as a consequence of the increased degradation of the *miTarget* by miR-31.

## DISCUSSION

miRNAs are fundamental building blocks of post-transcriptional control of gene expression and help to finely control genetic circuits and precisely regulate endogenous pathways. We observed that when a synthetic transcript is downregulated by an endogenous miRNA, a synthetic co-expressed mRNA is indirectly upregulated. Here, we combined mathematical modelling and experimental analysis to understand important mechanisms underlying the effect of miRNAs on resource allocation in mammalian cells, when their regulation is embedded in synthetic circuits. This understanding is instrumental to generate more detailed guidelines for an informed and rational design of gene circuits, and may also provide insights into endogenous gene regulation.

We had previously observed that exogenous genes delivered in mammalian cells compete for intracellular resources. This effect was observed, albeit to a different extent, regardless of genes amount and cell types (1).

Since miRNAs function at post-transcriptional level, we developed a mathematical model, MIRELLA, that considers effective reaction rates of biological processes to account for the availability of post-transcriptional shared resources. Specifically, the model considers one pool of mRNA translation resources and one pool of mRNA degradation resources, for simplicity represented by ribosomes and RNases, respectively (Figures 2A and 4A). Although the aforementioned biological processes are regulated by many molecular species, the key cause of resource competition in mammalian cells still remains an open question. Here we focused on ribosomes and RNases for their relevance in these processes and—relatively to ribosomes—for the possibility to experimentally measure them. This allows us to keep the model as simple as possible to study the role of two prime contributors—that is ribosomes and RNases—in gene expression burden. We anticipate that other molecular species could contribute to shaping gene expression as a result of resource competition, such as tRNAs for rare codons in mRNA translation. In this respect, our resource-aware modelling framework can be extended to capture more complex scenarios, for example, by including the resource competition for shared tRNAs in the model's equations. Moreover, our context-aware theory paves the way towards a holistic understanding of cell biology (30) instrumental to engineer more sophisticated biomolecular circuits that can maximise the global system's output (e.g. a protein or a compound of interest) by operating synergistically with the cell physiology (31)

The experimental setting is based on two fluorescent reporters driven by constitutive promoters, one regulated by an endogenous miRNA (*miTarget*) and the other lacking regulation, as a proxy for resources availability (*capacity monitor*). This design is simple and is meant to quickly appreciate differences in protein expression levels. The modelling framework suggested that miRNA regulation has two major consequences on resource allocation. One is a queueing effect on the RNA degradation pathway as the result of the strong miRNA-mediated slicing of *miTarget* transcripts. The other is an increased amount of ribosomes becoming available for translating other transcripts.

Synthetic networks that embed miRNA regulation typically employ perfectly complementary TS to maximise the fold change expression of the target gene (18), and it has been generally observed that the abundance of miRNAs with respect to their target affects genetic circuits functionality (32). It was previously proposed that a significant mRNA degradation by miRNA may lead to a ‘queueing effect’ for the degradation of other mRNAs, decreasing the effective mRNA decay rate (33). We validated this hypothesis by quantifying the mRNA decay of two endogenous genes, CCNA2 and eIF4E, which were previously shown to be impacted by the burden imposed by the expression of synthetic circuits (1). We observed that upon transcription-inhibition treatment with DRB, CCNA2 and eIF4E are degraded at a slower pace in the samples expressing *miTarget* as compared to the control lacking the TS. These results suggest that an accumulation of other mRNAs contributes to increased cognate protein levels. This finding may be useful for circuit design considerations, particularly when such systems are devised to study endogenous processes or pathways.

Our experimental findings suggest that ribosomes reallocate upon miRNA regulation and that this effect is more pronounced when miRNA-TS are located in the 5’UTR of the target gene. We speculate that the proximity of TS to the AUG may prevent ribosomes from binding due to steric hindrance, effectively reducing the translation of the transcript. It was previously observed that miRNA-RISC may decrease the rate of translation initiation (25). One mechanism reported that Argonaute proteins, along with miRNA and cognate targets accumulate in P-bodies in a miRNA-dependent manner (23,24,34,35), increasing the amount of ribosome-free mRNA and free ribosomes. Our model also suggested that the downregulation of the *miTarget* would result in more free ribosomes. Therefore, we performed polysome profiling to observe changes in the co-sedimentation profiles of transcripts with ribosomes in polysomes. Of note, a modified co-sedimentation profile of the *capacity monitor*, with a shift towards heavier gradient fractions (9–10, Figure 3C) in the 5’UTR-TS condition was expected. In contrast, the altered profile of the *miTarget* was surprising. In contrast, a previous study reported that translation repression by Let-7 miRNA in mammalian cells resulted in a shift of the mRNA target to lighter fractions in polysome gradients (24). We speculate that our system differs in TS location (5’ versus 3’UTR) and complementarity (perfect *vs* non-perfect) from the abovementioned study. Therefore, since there is a major effect of *miTarget* degradation due to the endonuclease activity of the miRISC complex, there is an enrichment of the remaining transcripts with the available ribosomes, including the *miTarget* transcripts escaping the miRNA activity. This speculation was supported also by our MIRELLA model. Regardless of *where* ribosomes reallocate, it is important to consider this secondary effect of miRNA regulation. The primary function of miRNAs is the downregulation of the target genes, and recently other properties of miRNA were demonstrated, such as buffering gene expression against noise or external inducer concentration (10–12). Our results indicate further hidden secondary effects that could play a role in the general homeostasis of protein expression. However, proving this idea in an endogenous system is challenging since miRNAs are embedded in intricate networks that do not allow to untangle single effects of miRNA regulation easily. In future studies, it could be interesting to analyse the impact of miRNAs on resource allocation as related to the number of ribosomes sequestered by the *miTarget*. This could be achieved by placing internal ribosome entry sites (IRES) in the target transcript and observing the effect on the *capacity monitor* once the miRNA activity is impaired (e.g. by miRNA-specific inhibitors).

Resource competition can dramatically affect circuits behaviour and cellular physiology. Conversely, burden mitigation can speed up the cumbersome design-build-test cycle and open up avenues for the predictive design and engineering of more reliable gene constructs in mammalian cells. In this respect, our study provides the scientific community with a necessary understanding for robustly and effectively building genetic devices and biomolecular controllers—for example, iFFL architectures based on miRNAs (1,36)—that rely on precise quantitative assessment of molecular species. Our study also highlights that the use of appropriate experimental controls for a desired genetic circuit architecture, along with appropriate computational tools may be key to avoid results misinterpretation and to correctly predict the overall outcome of the synthetic device. Moreover, our results contribute to compelling insights into primary and secondary regulatory effects of miRNA action with implications for basic science and for industrial and medical biotechnology.

## DATA AVAILABILITY

The source code that supports the findings of this study is available publicly from GitHub at https://github.com/giansimone/MIRELLA/ (DOI: 10.6084/m9.figshare.21725387). The data underlying this article are available in the article and in its online supplementary material (SourceData).

## Supplementary Material

gkad151_Supplemental_File

## Notes

Present address: Department of Biosystems Science and Engineering (D-BSSE), ETH Zürich, Mattenstrasse 26, Basel 4058, Switzerland
