# Supplementary material for: MIRELLA: a mathematical model explains the effect of microRNA-mediated synthetic genes regulation on intracellular resource allocation
Source: Nucleic Acids Res. 2023 Mar 13;51(7):3452–64. doi: 10.1093/nar/gkad151 (PMC10123119; doi:10.1093/nar/gkad151)
Supplement: gkad151_Supplemental_File [file gkad151_supplemental_file.pdf]

Supplementary Information

MIRELLA: a mathematical model explains the effect of MIcroRNA-mediated synthetic genes REgulation on intracellular resource aLLocAtion

Authors:

Federica Cella[§1,7], Giansimone Perrino[§3], Fabiana Tedeschi[1,2], Gabriella Viero[4], Carla Bosia[5,6], Guy-Bart Stan*[3], Velia Siciliano*[1].

[1] Istituto Italiano di Tecnologia-IIT, Largo Barsanti e Matteucci, Naples ITA
[2] University of Naples Federico II, Naples ITA
[3] Department of Bioengineering and Centre of Excellence in Synthetic Biology, Imperial College London, U.K.
[4] Institute of Biophysics, CNR Trento, Povo, ITA
[5] Department of Applied Science and Technology, Politecnico di Torino, Torino, ITA
[6] Italian Institute for Genomic Medicine, c/o IRCCS, Candiolo, Italy.
[§] Equal contribution
[7] Present address: Department of Biosystems Science and Engineering (D-BSSE), ETH Zürich, Mattenstrasse 26, Basel, 4058, Switzerland

* Correspondence should be sent to Guy-Bart Stan (g.stan@imperial.ac.uk) and Velia Siciliano (velia.siciliano@iit.it)

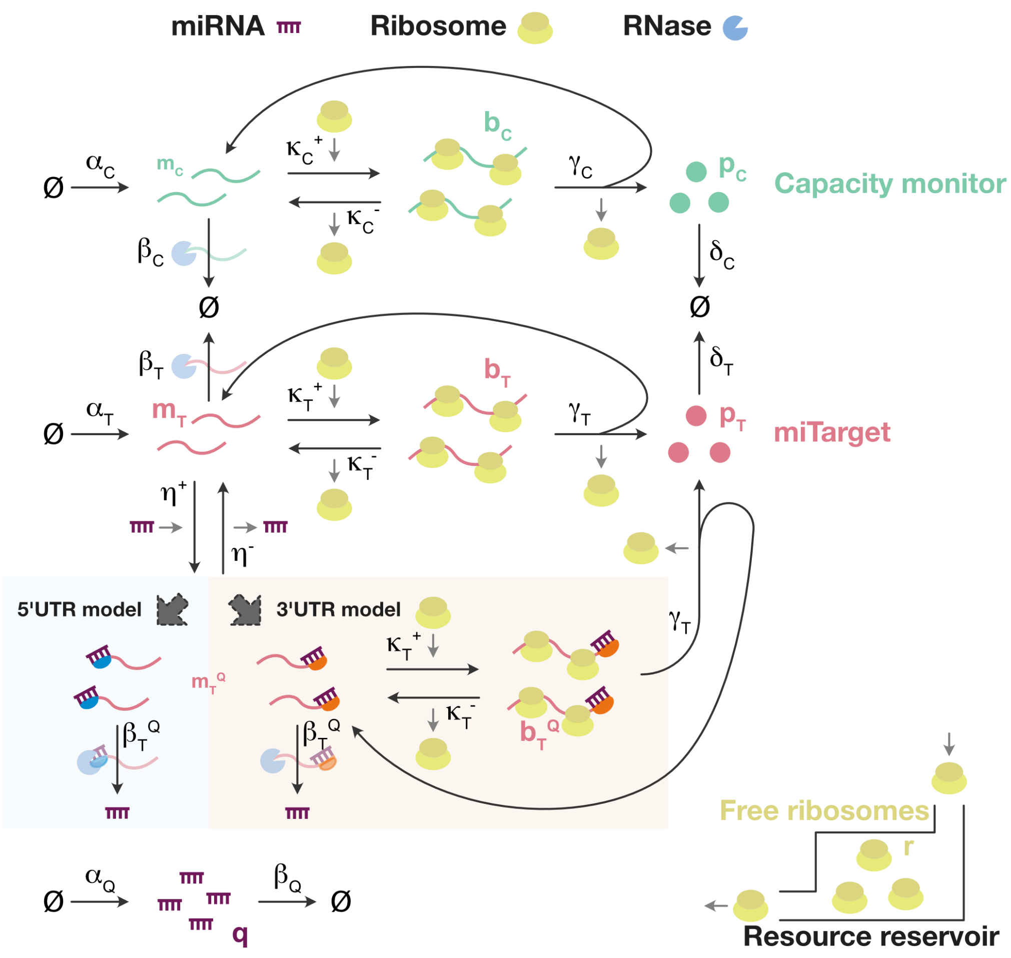

Supplementary Figure 1. Extended graphical representation of the model used to study the reallocation of the translational resources (ribosomes). Full schematics of the model used to study ribosomes reallocation caused by miRNA-driven regulation of an exogenous two-gene circuit in H1299 cells. For each of the two exogenous genes, the model captures the essential features of transcription, translation, degradation, and interactions between genes and ribosomes. The shared cellular resource pool for RNA degradation (RNases) is here considered unlimited. An exhaustive description of the model can be found in **Supplementary Notes 1-3**. All the molecular species captured in the model are listed in **Supplementary Table 4**, whilst all the model parameters – including the numerical values used for the simulations – are summarised in **Supplementary Table 5**.

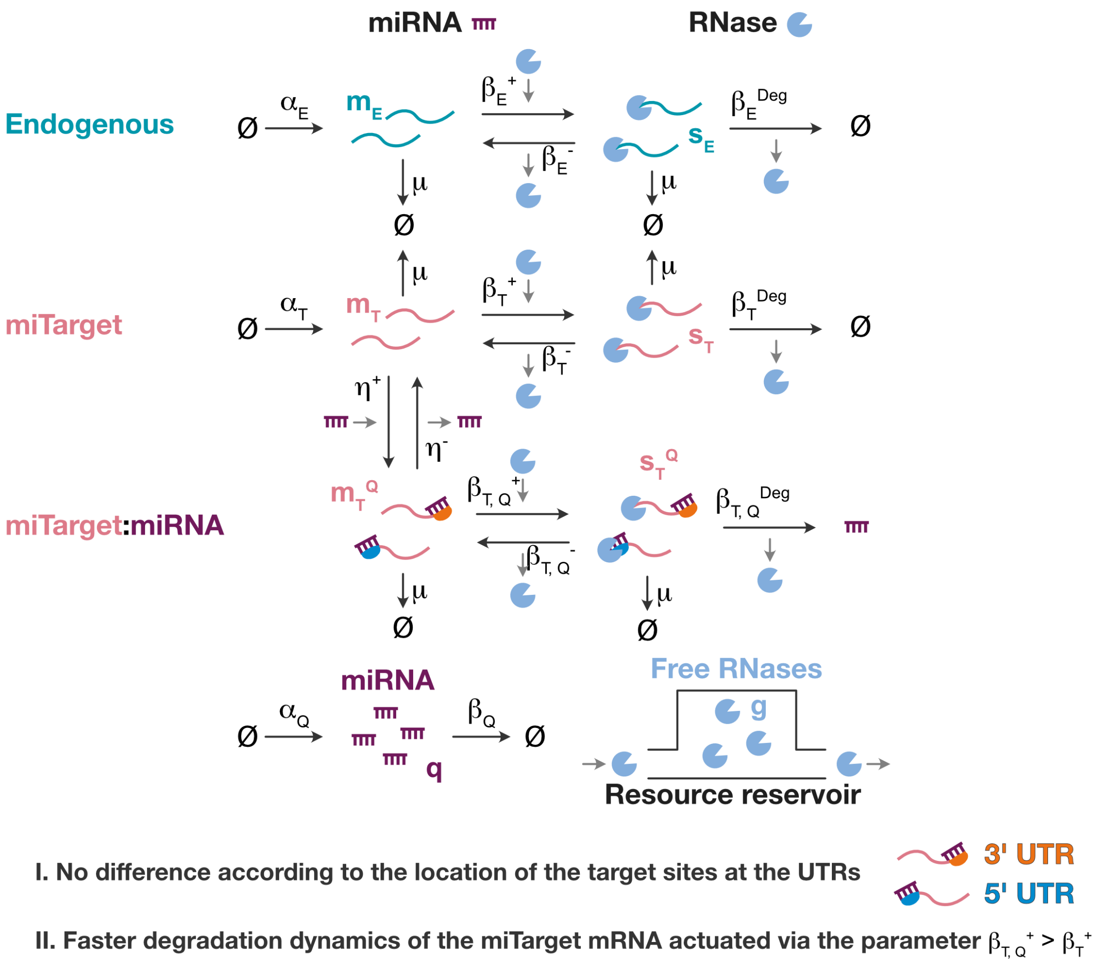

Supplementary Figure 2. Extended graphical representation of the model used to study the reallocation of the degradation resources (e.g. RNases). The model depicted in **Fig. 2a** and **Supplementary Fig. 1** is extended to include a finite pool of RNases. With this assumption, the model recapitulates the essential features of transcription, degradation, and interactions between genes and RNases. An exhaustive description of the model can be found in **Supplementary Note 5**. All the molecular species captured in the model are listed in **Supplementary Table 6**, whilst all the model parameters – including the numerical values used for the simulations – are summarised in **Supplementary Table 7**.

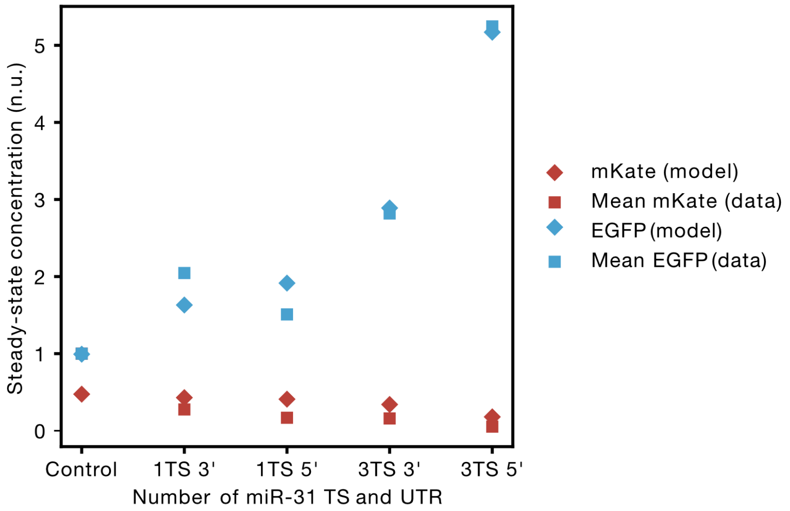

Supplementary Figure 3. Model fitting related to the experimental data shown in Fig. 2b. The ODE framework shown in **Fig. 2a** was applied to model the steady-state experimental data (squares) shown in **Fig. 2b** (**Methods**). A description of the model can be found in **Supplementary Notes 1-3**. All the molecular species captured in the model are listed in **Supplementary Table 4**. The experimental data are presented as mean values.

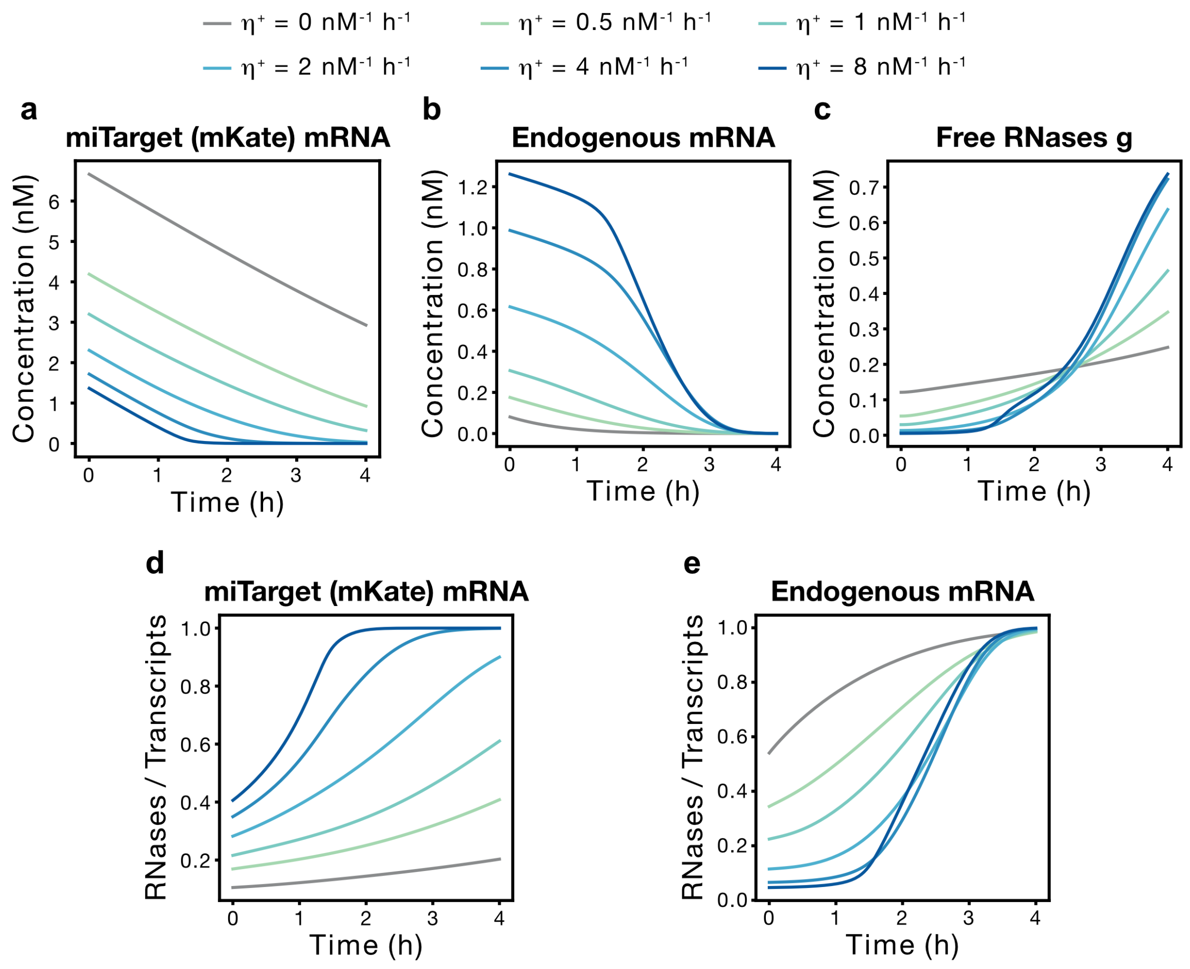

Supplementary Figure 4. Extended simulation data for Fig. 4b. (**a, b**) Predicted mRNA degradation profiles for the *miTarget* (**a**) and the endogenous gene (**b**) upon halting of transcription for different values of the miRNA binding constant η^+. (**c**) Predicted reallocation profiles for the RNases (i.e., degrading resource) for different values of the miRNA binding constant η^+. (**d, e**) Predicted degrading resource densities (i.e., RNases/transcripts) for the *miTarget* (**d**) and the endogenous gene (**e**). Each colour represents a different value for the miRNA binding constant η^+. A description of the model can be found in **Supplementary Note 5**. All the molecular species captured in the model are listed in **Supplementary Table 6**, whilst all the model parameters – including the numerical values used for the simulations – are summarised in **Supplementary Table 7**.

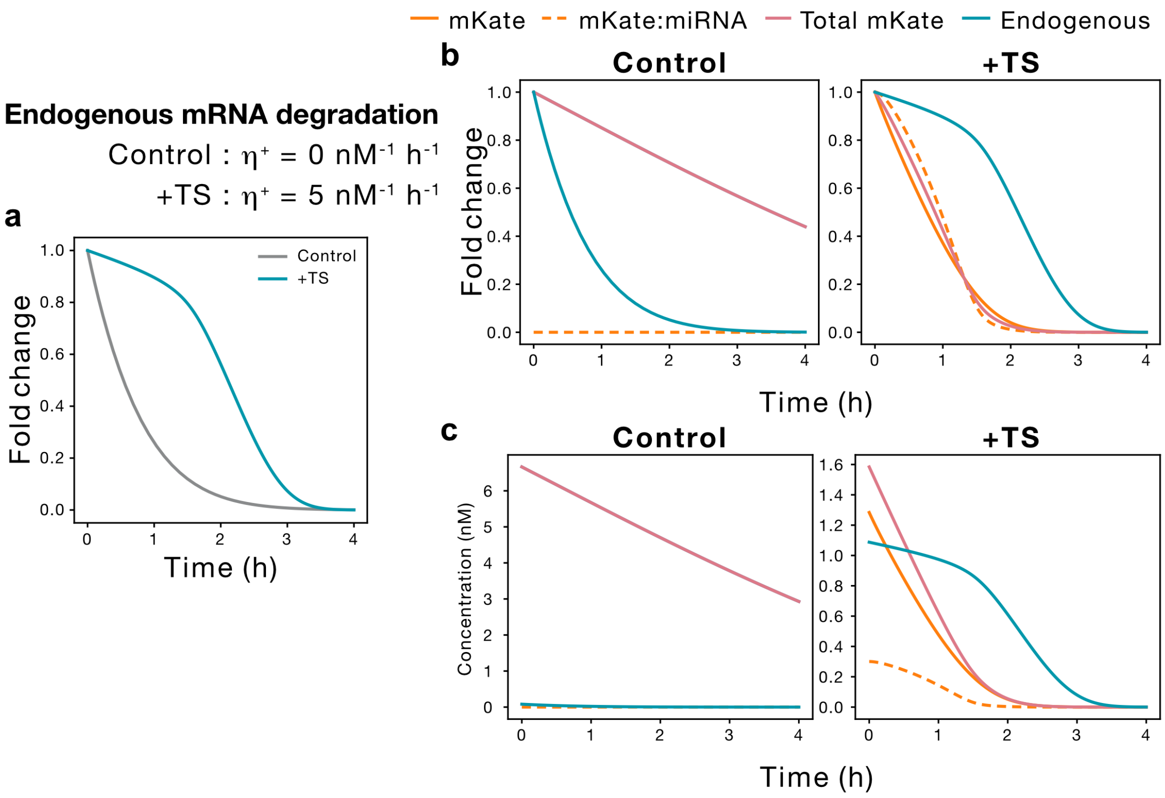

Supplementary Figure 5. Example of an endogenous mRNA decay with and without reallocation of the shared degrading resource pool. (**a**) Predicted fold change for the mRNA degradation profiles of an endogenous gene upon halting of transcription with (+TS) and without (Control) miR-TS at the UTRs of the *miTarget* gene. (**b**) Predicted fold change for the mRNA decay profiles of all mRNA species involved in the model upon halting of transcription with and without miR-TS at the UTRs of the *miTarget* (mKate) gene. (**c**) Predicted mRNA expression levels for all mRNA species involved in the model upon halting of transcription with and without miR-TS at the UTRs of the *miTarget* (mKate) gene. The model predicts an impairment of the endogenous mRNA decay upon miRNA-driven regulation that is caused by a reallocation of the RNases on the exogenous *miTarget* gene. A description of the model can be found in **Supplementary Note 5**. All the molecular species captured in the model are listed in **Supplementary Table 6**, whilst all the model parameters – including the numerical values used for the simulations – are summarised in **Supplementary Table 7**.

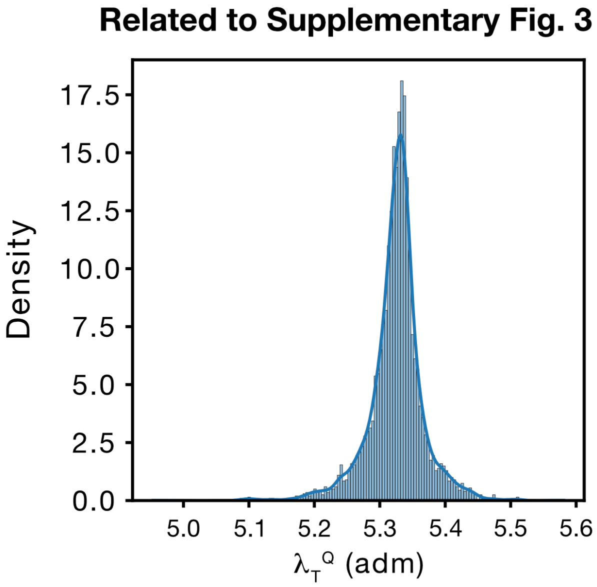

Supplementary Figure 6. Histogram and probability density function for the model parameter λ_T^Q. The distribution of the inferred numerical values for the model parameter λ_T^Q was obtained by repeating the model fitting (**Methods**) 5,000 times (see an instance of the model fitting in **Supplementary Fig. 3**). Mean value calculated from the probability density function: $\overline{\lambda}_T^Q$ = 5.325.

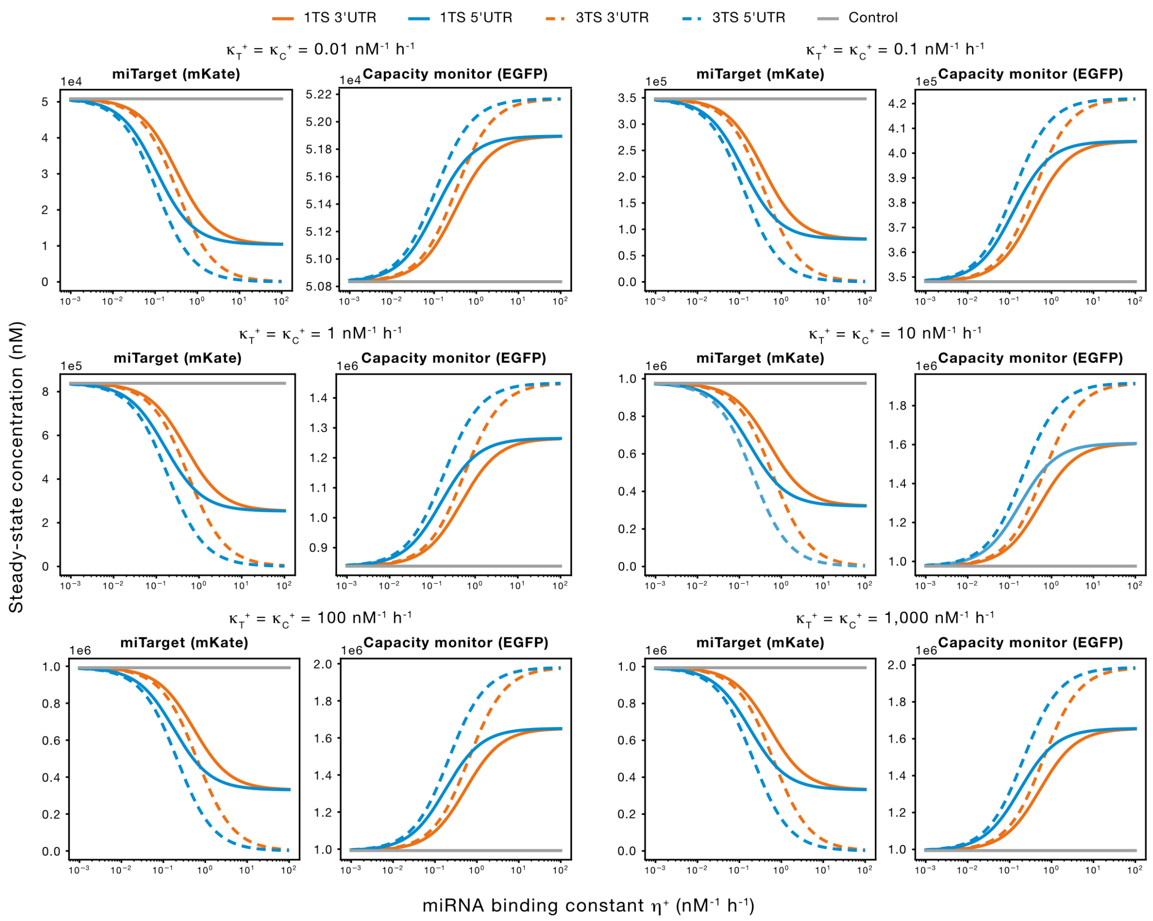

Supplementary Figure 7. The simulated qualitative trend of the protein expression levels does not change when varying κ_T^+ and κ_C^+. The ODE model illustrated in **Fig. 2a** was simulated using different values for the association constants κ_T^+ and κ_C^+ to understand how the miRNA-driven regulation changes the protein expression levels for the *miTarget* (mKate) and the *capacity monitor* (EGFP). The two association constants were set to the same value (i.e., $\kappa_T^+ = \kappa_C^+$) since the two transcripts have similar RNA sequences, hence the binding rate constants κ_T^+ and κ_C^+ may only slightly differ from each other. Numerical simulations show that the qualitative trend of the protein expression levels does not change when varying κ_T^+ and κ_C^+, but the absolute protein levels do. Each colour represents a different design condition that depends on the location and the number of the miRNA target sites at the UTRs of the *miTarget* gene. A description of the model can be found in **Supplementary Notes 1-3**. All the molecular species captured in the model are listed in **Supplementary Table 4**, whilst all the model parameters – including the numerical values used for the simulations – are summarised in **Supplementary Table 5**.

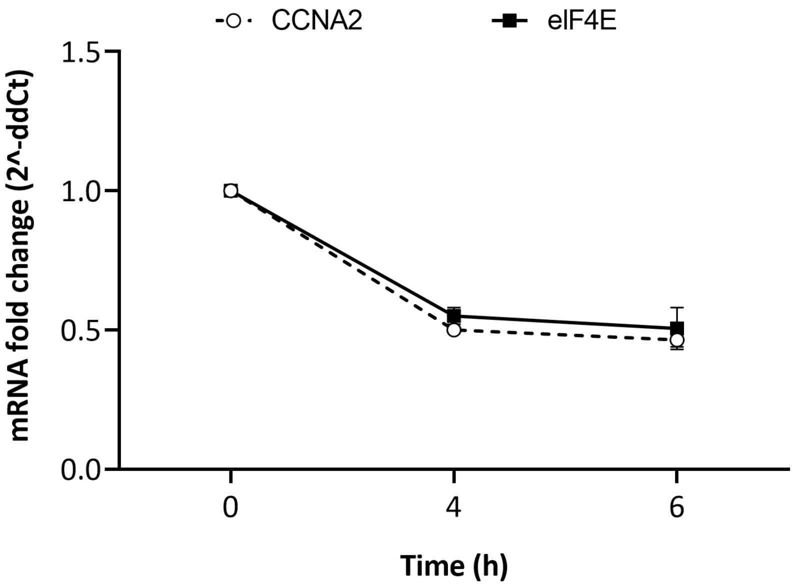

Supplementary Figure 8. CCNA2 and eIF4E half-life in H1299 cells treated with DRB.
mRNA half-life of CCNA2 and eIF4E were measured up to 6 hours post-DRB treatment in wild type cells. All data are plotted as mean +/- SE. SE: standard error. N=2 replicates.

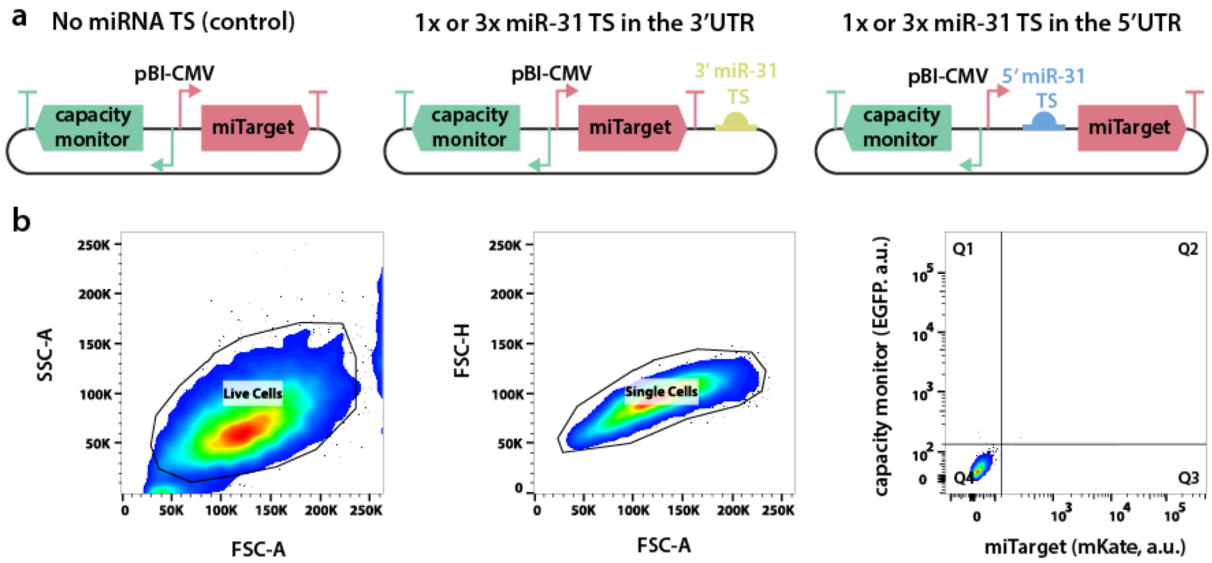

Supplementary Figure 9. Plasmids design and gating strategy for flow cytometry experiments. (a) All plasmids encode for two fluorescent proteins, namely the *capacity monitor* (EGFP) and *miTarget* (mKate) proteins, under the regulation of a bidirectional CMV promoter (pBI-CMV). No miRNA TS (control): plasmid without target sites for miRNA-31. Plasmids encoding for 1x or 3x miR-31 target sites (TS) either in the 3'UTR or 5'UTR of the *miTarget* are used to study the effect of miRNA on resource reallocation. **(b)** Gating strategy, example on non-transfected cells to set the positive threshold for each fluorescence. Left, the recorded events were gated first in the FSC-A vs SSC-A channels to select the Live Cells population. Centre, the Live Cells population was then gated in the FSC-A vs FSC-H channels to select the single-cell population. Right, the geometric mean of cells in the Q2 quadrant (double positive) was pulled out.

Supplementary Table 1. Transfection Tables.

Figure 3c-d

	pBI-F3G	pBI-H1G/H3G/H5G/H7G	pEmpty
Control	2.9 µg		5.6 µg
miR-31 TS		2.9 µg	5.6 µg
Reagent/cells			
Optimem	1450 µL		
Lipofectamine 3000	21.75 µL		
P3000	29 µL		
H1299	3480000		

Figure 4c-d

	pBI-G	pL-A1	pH-3/pH-7	pEmpty
Control	50 ng	50 ng		200 ng
miR-31 TS	50 ng		50 ng	200 ng
Reagent/cells				
Optimem	50 µL			
Lipofectamine 3000	0.75 µL			
P3000	1 µL			
H1299	150000			

Supplementary Table 2. List of plasmids used for this study.

Fig.	Short plasmid name	Full plasmid name	Parts from	GenBank accession code
Fig. 3c-d	pBI-F3G	pBI-CMV1_EGFP_mKate	Clontech 631630	MT891342
Fig. 3c-d	pBI-H1G	pBI-CMV1_EGFP_mKate_1xmiR31TS5'	Clontech 631630	MT891344
Fig. 3c-d	pBI-H3G	pBI-CMV1_EGFP_mKate_3xmiR31TS5'	Clontech 631630	MT891345
Fig. 3c-d	pBI-H5G	pBI-CMV1_EGFP_mKate_1xmiR31TS3'	Clontech 631630	MT891346
Fig. 3c-d	pBI-H7G	pBI-CMV1_EGFP_mKate_3xmiR31TS3'	Clontech 631630	MT891347
Fig. 4c-d	pBI-G	pBI-CMV1_EGFP	Clontech 631630	MT891343
Fig. 4c-d	pL-A1	pT-GTW6-CMV-mKate	(1)	MT891367
Fig. 4c-d	pH-3	pT-GTW6-CMV-mKate_3xmiR31TS5'	(1)	MT891350
Fig. 4c-d	pH-7	pT-GTW6-CMV-mKate_3xmiR31TS3'	(2)	MT891353

Supplementary Table 3. qPCR primers used in this study.

Primer	Function	Sequence (5'-3')
F7	Forward primer for mKate amplification	GGTGTCTAAGGGCGAAGAGC
F8	Reverse primer for mKate amplification	GCTGGTAGCCAGGATGTCGA
qPCR-EGFP-F	Forward primer for EGFP amplification	AAGGGCATCGACTTCAAG
qPCR-EGFP-R	Reverse primer for EGFP amplification	TGCTTGTCGGCCATGATATG
qPCR-18S-F	Forward primer for 18S amplification	GCTTAATTTGACTCAACACGGGA
qPCR-18S-R	Reverse primer for 18S amplification	AGCTATCAATCTGTCAATCCTGTC
qPCR-GAPDH-F	Forward primer for GAPDH amplification	GAAGATGGTGATGGGATTTC
qPCR-GAPDH-R	Reverse primer for GAPDH amplification	GAAGTTGAAGGTCGGAGT
qPCR-CCNA2-F	Forward primer for CCNA2 amplification	GGGACAAAGCTGGCCTGAATC
qPCR-CCNA2-R	Reverse primer for CCNA2 amplification	AGGTAGGTCTGGTGAAGGTCC
qPCR-eIF4E-F	Forward primer for eIF4E amplification	AGAACAGATGGGCACTCTGX
qPCR-eIF4E-R	Reverse primer for eIF4E amplification	TGAGTAGTCACAGCCAGGC

Supplementary Table 4. Molecular species simulated in the model to study the reallocation of translational resources. Related to Supplementary Notes 1-4.

**Description**	**Species**	**Units**
miTarget mRNA	m_T	nM
Translating *miTarget* mRNA	b_T	nM
miTarget:miRNA mRNA complex	m_T^Q	nM
Translating *miTarget:miRNA* mRNA complex	b_T^Q	nM
miRNA	q	nM
Capacity monitor mRNA	m_C	nM
Translating *capacity monitor* mRNA	b_C	nM
miTarget protein (mKate)	p_T	nM
Capacity monitor protein (EGFP)	p_C	nM
Free ribosomes	r	nM

Supplementary Table 5. Numerical parameters used to simulate the model to study the reallocation of translational resources. Related to Supplementary Notes 1-4.

Description	Parameter	Value	Units
miTarget transcription rate constant	α_T	1	nM h^{-1}
miRNA binding constant	η^+	[0.001, 100]	nM^{-1} h^{-1}
miRNA dissociation constant	η^-	0	h^{-1}
miTarget mRNA degradation rate constant	β_T	0.231	h^{-1}
miTarget:miRNA mRNA degradation rate constant	β_T^Q	1.155	h^{-1}
miTarget translation rate constant	γ_T	53	h^{-1}
miTarget ribosomal scaling factor	n_T	33	Unitless
Association constant between a *miTarget* mRNA and a ribosome	κ_T^+	1	nM^{-1} h^{-1}
Dissociation constant of the *miTarget* translating complex	κ_T^-	0	h^{-1}
miTarget protein degradation rate constant	δ_T	0.027	h^{-1}
miRNA transcription rate constant	α_Q	0.05	nM h^{-1}
miRNA degradation rate constant	β_Q	0.069	h^{-1}
Capacity monitor transcription rate constant	α_C	1	nM h^{-1}
Capacity monitor translation rate constant	γ_C	53	h^{-1}
Capacity monitor ribosomal scaling factor	n_C	33	Unitless
Association constant between a the *Capacity monitor* mRNA and a ribosome	κ_C^+	1	nM^{-1} h^{-1}
Dissociation constant of a *Capacity monitor* translating complex	κ_C^-	0	h^{-1}
Capacity monitor protein degradation rate constant	δ_C	0.027	h^{-1}
Total ribosomes available in the model	r^{Total}	1000	nM

Supplementary Table 6. Molecular species simulated in the model to study the reallocation of degradation resources. Related to Supplementary Note 5.

Description	Species	Units
miTarget mRNA	m_T	nM
Degrading *miTarget:RNase* complex	s_T	nM
miTarget:miRNA mRNA complex	m_T^Q	nM
Degrading *miTarget:miRNA:RNase* complex	s_T^Q	nM
miRNA	q	nM
Endogenous mRNA	m_E	nM
Degrading E*ndogenous:RNase* complex	s_E	nM
Free RNases	g	nM

Supplementary Table 7. Numerical parameters used to simulate the model to study the reallocation of degradation resources. Related to Supplementary Note 5.

Description	Parameter	Value	Units
miTarget transcription rate constant	α_T	1	nM h^{-1}
miRNA binding constant	η^+	{0, 0.5, 1, 2, 4, 8}	nM^{-1} h^{-1}
miRNA dissociation constant	η^-	0	h^{-1}
Association constant between a *miTarget* mRNA and an RNase	β_T^+	1	nM^{-1} h^{-1}
Dissociation constant of a *miTarget:RNase* complex	β_T^-	0	h^{-1}
miTarget:RNase complex degradation rate constant	β_T^{Deg}	1	h^{-1}
Association constant between a *miTarget:miRNA* complex and an RNase	$\beta_{T,Q}^+$	500	nM^{-1} h^{-1}
Dissociation constant of *miTarget:miRNA:RNase* complex	$\beta_{T,Q}^-$	0	h^{-1}
miTarget:miRNA:RNase complex degradation rate constant	$\beta_{T,Q}^{Deg}$	1	h^{-1}
miRNA transcription rate constant	α_Q	0.05	nM h^{-1}
miRNA degradation rate constant	β_Q	0.069	h^{-1}
Endogenous transcription rate constant	α_E	0.1	nM h^{-1}
Association constant between an endogenous mRNA and an RNase	β_E^+	10	nM^{-1} h^{-1}
Dissociation constant of the *Endogenous:RNase* complex	β_E^-	0	h^{-1}
Endogenous:RNase complex degradation rate constant	β_E^{Deg}	1	h^{-1}
Total RNase available in the model	g^{Total}	1	nM
Cell growth rate	μ	0.029	h^{-1}

Supplementary Note 1. Resource-aware model to study reallocation of translational resources.

In what follows we describe the resource-aware deterministic model used to study the reallocation of translational resources (i.e., ribosomes) as introduced in **Fig. 2** and **Fig. 3**. The deterministic model is based on an existing resource-aware modelling framework (2) and considers ribosomes as the main cellular resources shared between co-expressed genes. Our model replaces reaction rates that involve shared translational resources with effective reaction rates that account for the availability of these resources according to the overall demand from co-expressed genes as shown in **Fig. 1b**. The key assumptions underlying the formulation of the model equations are listed and discussed in the main text. Briefly, we make the following assumptions:

1. The shared cellular resource pool for translation (ribosomes) is considered finite and constant.
2. The shared cellular resource pool for RNA degradation (RNases) is considered unlimited.
3. Shared transcriptional resource pools (e.g., RNA polymerases) are not explicitly considered in the model. Such an assumption does not alter the findings of our study as shown in **Supplementary Note 2**.

To derive the deterministic model, we apply the law of mass action to the biochemical reactions shown in **Supplementary Fig. 1**. Hence, we obtain the following set of ordinary differential equations (ODE):

$$\frac{dm_T}{dt} = \alpha_T - \kappa_T^+ \, m_T r + \left(\kappa_T^- + \gamma_T\right) b_T + \eta^- m_T^Q - \left(\beta_T + \eta^+ q\right) m_T$$

$$\frac{db_T}{dt} = \kappa_T^+ \, m_T r - \left(\kappa_T^- + \gamma_T\right) b_T$$

$$\frac{dm_T^Q}{dt} = \eta^+ q \, m_T - \left(\beta_T^Q + \eta^-\right) m_T^Q - \sigma \cdot \kappa_T^+ \, m_T^Q r + \sigma \cdot \left(\kappa_T^- + \gamma_T\right) b_T^Q$$

$$\frac{db_T^Q}{dt} = \kappa_T^+ \, m_T^Q r - \left(\kappa_T^- + \gamma_T\right) b_T^Q \qquad \text{(applies only to the 3' UTR model)}$$

$$\frac{dq}{dt} = \alpha_Q + \left(\beta_T^Q + \eta^-\right) m_T^Q - \left(\beta_Q + \eta^+ \, m_T\right) q$$

$$\frac{dm_C}{dt} = \alpha_C - \beta_C \, m_C - \kappa_C^+ \, m_C r + \left(\kappa_C^- + \gamma_C\right) b_C$$

$$\frac{db_C}{dt} = \kappa_C^+ \, m_C r - \left(\kappa_C^- + \gamma_C\right) b_C$$

$$\frac{dp_T}{dt} = \gamma_T \left(b_T + \sigma \cdot b_T^Q \right) - \delta_T \, p_T$$

$$\frac{dp_C}{dt} = \gamma_C\ b_C - \delta_C\ p_C$$

where the boolean parameter $\sigma\ \in\ \{0,\ 1\}$ captures the location of the miR-TS at the UTRs. Specifically, σ is set to 1 if the miR-TS are at the 3' UTR, otherwise σ is set to 0. When considering miR-TS at the 3' UTR (i.e., when $\sigma\ =\ 1$), the model captures the additional inhibition of translation by the *miTarget:miRNA* mRNA complex. A list of all the molecular species modelled is reported in **Supplementary Table 4**, whilst a list of all the model parameters – including the numerical values used to simulate the model (see **Supplementary Note 6** for the model parameterisation) – is reported in **Supplementary Table 5**.

Assuming that the translating ribosomal complexes b_T, b_C, and b_T^Q (the latter being considered only in the 3' UTR model) reach their steady state faster than the other molecular species involved in the model, we can make a quasi-steady-state approximation (QSSA) for the dynamics of the translating ribosomal complexes in our proposed resource-aware model. Such an assumption is biologically reasonable since a ribosome typically binds to a transcript with a timescale faster than that associated with the production and degradation of mRNA and protein species. Therefore, assuming a QSSA for the translating ribosomal complexes concentrations, we obtain the following steady-state concentrations – which we denote with a bar:

$$\frac{db_T}{dt} \approx\ 0 \Rightarrow\ \overline{b}_T = \frac{\kappa_T^+}{\kappa_T^- + \gamma_T}\, m_T\, r\ = \frac{1}{\kappa_T}\, m_T\, r$$

$$\frac{db_T^Q}{dt} \approx\ 0 \Rightarrow \overline{b}_T^Q = \frac{\kappa_T^+}{\kappa_T^- + \gamma_T}\, m_T^Q\, r\ = \frac{1}{\kappa_T}\, m_T^Q\, r \qquad \text{(applies only to the in 3' UTR model)}$$

$$\frac{db_C}{dt} \approx\ 0 \Rightarrow\ \overline{b}_C = \frac{\kappa_C^+}{\kappa_C^- + \gamma_C}\, m_C\, r\ = \frac{1}{\kappa_C}\, m_C\, r$$

where the lumped parameters κ_T and κ_C denote the effective dissociation constants between the mRNA species and the ribosome species. Substituting these quasi-steady-state concentrations in the resource-aware model yields the reduced ODE model:

$$\frac{dm_T}{dt} = \alpha_T + \eta^-\ m_T^Q - \left(\beta_T + \eta^+\ q\right) m_T$$

$$\frac{dm_T^Q}{dt} = \eta^+\ q\ \ m_T - \left(\beta_T^Q + \eta^-\right) m_T^Q$$

$$\frac{dq}{dt} = \alpha_Q + \left(\beta_T^Q + \eta^-\right) m_T^Q - \left(\beta_Q + \eta^+\ \ m_T\right) q$$

$$\frac{dm_C}{dt} = \alpha_C - \beta_C\ m_C$$

$$\frac{dp_T}{dt} = \gamma_T \frac{m_T + \sigma\, m_T^Q}{\kappa_T} r - \delta_T\, p_T$$

$$\frac{dp_C}{dt} = \gamma_C \frac{m_C}{\kappa_C} r - \delta_C\, p_C$$

Assuming a limited constant amount of available translational resources (r^{Total}), we can apply a resource conservation law on the total concentration of translational resources:

$$r^{Total} = r + \bar{b}_T + \bar{b}_T^Q + \bar{b}_C$$

Substituting the steady-state concentrations of the translating ribosomal complexes in the previous equation yields the concentration of free ribosomes:

$$r = \frac{r^{Total}}{1 + \frac{m_T + \sigma\, m_T^Q}{\kappa_T} + \frac{m_C}{\kappa_C}}$$

Substituting the concentration of free ribosomes in the reduced model yields the resource-aware ODE model (**Fig. 2a**):

$$\frac{dm_T}{dt} = \alpha_T + \eta^- m_T^Q - \left(\beta_T + \eta^+ q\right) m_T$$

$$\frac{dm_T^Q}{dt} = \eta^+ q\ m_T - \left(\beta_T^Q + \eta^-\right) m_T^Q$$

$$\frac{dq}{dt} = \alpha_Q + \left(\beta_T^Q + \eta^-\right) m_T^Q - \left(\beta_Q + \eta^+\ m_T\right) q$$

$$\frac{dm_C}{dt} = \alpha_C - \beta_C\ m_C$$

$$\frac{dp_T}{dt} = \gamma_T^{Eff} + \gamma_{T,Q}^{Eff} - \delta_T\ p_T$$

$$\frac{dp_C}{dt} = \gamma_C^{Eff} - \delta_C\ p_C$$

where the effective translation rate constants are defined as:

$$\gamma_T^{Eff} = \gamma_T \cdot \frac{\frac{m_T}{\kappa_T}}{1 + \frac{m_T + \sigma\, m_T^Q}{\kappa_T} + \frac{m_C}{\kappa_C}} \cdot r^{Total} = \gamma_T \cdot \frac{\frac{m_T}{\kappa_T}}{1 + \frac{m_C}{\kappa_C} + \frac{\sigma\, m_T^Q}{\kappa_T} + \frac{m_T}{\kappa_T}} \cdot r^{Total} = \gamma_T \cdot f_T\left(m_T, \sigma\, m_T^Q, m_C\right) \cdot r^{Total}$$

$$\gamma_{T,Q}^{Eff} = \gamma_T \cdot \frac{\frac{\sigma\ m_T^Q}{\kappa_T}}{1+\frac{m_T+\sigma\ m_T^Q}{\kappa_T}+\frac{m_C}{\kappa_C}} \cdot r^{Total} = \gamma_T \cdot \frac{\frac{\sigma\ m_T^Q}{\kappa_T}}{1+\frac{m_C}{\kappa_C}+\frac{m_T}{\kappa_T}+\frac{\sigma\ m_T^Q}{\kappa_T}} \cdot r^{Total} = \gamma_T \cdot f_{T,Q}\left(m_T,\ \sigma\ m_T^Q,\ m_C\right) \cdot r^{Total}$$

$$\gamma_{C}^{Eff} = \gamma_C \cdot \frac{\frac{m_C}{\kappa_C}}{1+\frac{m_T+\sigma\ m_T^Q}{\kappa_T}+\frac{m_C}{\kappa_C}} \cdot r^{Total} = \gamma_C \cdot \frac{\frac{m_C}{\kappa_C}}{1+\frac{m_T+\sigma\ m_T^Q}{\kappa_T}+\frac{m_C}{\kappa_C}} \cdot r^{Total} = \gamma_T \cdot f_{C}\left(m_T,\ \sigma\ m_T^Q,\ m_C\right) \cdot r^{Total}$$

These lumped parameters represent the core of the resource-aware model and reflect the dependency of the mRNA translation rates on the effective availability of translational resources in the cell. This dependency is captured via the regulatory functions f_T, $f_{T,Q}$, and f_C. The use of effective rates that take into account availability of shared cellular resources is conceptually illustrated in **Fig. 1b**.

The parameters used to simulate the model are summarised in **Supplementary Table 5**.

Supplementary Note 2. Negligible effects of transcriptional burden in the study of post-transcriptional resources reallocation.

As we are focusing on post-transcriptional events, the model does not explicitly consider shared pools of transcriptional resources (**Supplementary Note 1**). The inclusion or not of a shared transcriptional resource pool does not affect the results and the conclusions since variations in transcriptional burden can be accounted for by a change in the transcription rate constants α_T, α_C, and α_Q.

Similarly to the derivation done in **Supplementary Note 1** for the resource-aware translation dynamics, we can augment the model to also consider a limited pool of shared transcriptional resources (e.g., RNA polymerases) and thus capture transcriptional burden via effective transcription rate constants. Let a_T, a_C, and a_Q be the DNA concentrations for the *miTarget* gene, the *Capacity monitor* gene, and the *miRNA*; respectively. Let h^{Total} and h denote the available amounts of total and free transcriptional resources, respectively. Then, the ODEs associated with the RNA species can be rewritten using the effective transcription rate constants α_T^{Eff} (*miTarget*), α_C^{Eff} (*Capacity monitor*), and α_Q^{Eff} (*miRNA*) as follows:

$$\frac{dm_T}{dt} = \alpha_T^{Eff} + \eta^- m_T^Q - \left(\beta_T + \eta^+ q\right) m_T$$

$$\frac{dm_T^Q}{dt} = \eta^+ q\ m_T - \left(\beta_T^Q + \eta^-\right) m_T^Q$$

$$\frac{dq}{dt} = \alpha_Q^{Eff} + \left(\beta_T^Q + \eta^-\right) m_T^Q - \left(\beta_Q + \eta^+\ m_T\right) q$$

$$\frac{dm_C}{dt} = \alpha_C^{Eff} - \beta_C\ m_C$$

where the effective transcription rate constants are defined as:

$$\alpha_T^{Eff} = \alpha_T \cdot \frac{\frac{a_T}{\nu_T}}{1+\frac{a_T}{\nu_T}+\frac{a_C}{\nu_C}+\frac{a_Q}{\nu_Q}} \cdot h^{Total} = \alpha_T \cdot f_T^H\left(a_T,\ a_C, a_Q\right) \cdot h^{Total}$$

$$\alpha_Q^{Eff} = \alpha_Q \cdot \frac{\frac{a_Q}{\nu_Q}}{1+\frac{a_T}{\nu_T}+\frac{a_C}{\nu_C}+\frac{a_Q}{\nu_Q}} \cdot h^{Total} = \alpha_Q \cdot f_Q^H\left(a_T,\ a_C, a_Q\right) \cdot h^{Total}$$

$$\alpha_C^{Eff} = \alpha_C \cdot \frac{\frac{a_C}{\nu_C}}{1+\frac{a_T}{\nu_T}+\frac{a_C}{\nu_C}+\frac{a_Q}{\nu_Q}} \cdot h^{Total} = \alpha_C \cdot f_C^H\left(a_T,\ a_C, a_Q\right) \cdot h^{Total}$$

and ν_T, ν_C, and ν_Q are the effective dissociation constants between a DNA strand and an RNA polymerase for the *miTarget* gene, the *Capacity monitor* gene, and the *miRNA*; respectively. These

lumped parameters α_T^{Eff}, α_C^{Eff}, and α_Q^{Eff} exclusively depend on the DNA concentrations (a_T, a_C, and a_Q). Therefore, the transcriptional burden may alter the transcriptional profiles of the mRNAs. Nevertheless, the miRNA-driven regulation doesn't affect the effective transcriptional rate constants, neither implicitly nor explicitly. Hence, the effective transcription rates can be considered constant within the scope of this study. Without loss of generality, the superscript "Eff" associated with the effective transcription rate constants is omitted to simplify notations throughout the study.

Supplementary Note 3. Embedding polysomes into the resource-aware model to study reallocation of translational resources.

The model derived in **Supplementary Note 1** considers a set of 1:1 stoichiometric reactions between a transcript and a ribosome. However, a single transcript can be simultaneously translated by a group of ribosomes forming a complex known as polysome. Similarly to what was done in a previous resource-aware framework (3), the model can implicitly account for this by rescaling a subset of the model parameters according to the ribosome footprint on a transcript (4, 5).
The key idea consists of considering a series of mRNA spots in lieu of a single mRNA spot per transcript. More specifically, to account for polysomes, the model considers for each transcript a fixed number of mRNA spots that depends on the ribosome footprint size. Hence, each ribosome can bind only to a single mRNA spot. To capture this behaviour, the mRNA transcript concentrations are artificially augmented by rescaling a subset of model parameters as follows:

$$\alpha_T' = n_T \alpha_T$$

$$\alpha_Q' = n_T \alpha_Q$$

$$\alpha_C' = n_C \alpha_C$$

$$\eta^{+}{}' = \frac{\eta^{+}}{n_T}$$

where n_T and n_C are the maximum ribosomal densities – as defined in **Supplementary note 4** – achievable for the *miTarget* and the *capacity monitor*, respectively. Without loss of generality, we omit the prime symbol in the previous parameters to simplify the notation. Although the miRNA does not undergo a translation process (by definition), the miRNA transcription rate constant needs to be rescaled in order to maintain consistency between the miRNA concentration and the *miTarget* mRNA spots (i.e., a 1:1 stoichiometric ratio).
Under the previous considerations, the corresponding ODE model becomes:

$$\frac{dm_T}{dt} = n_T \alpha_T + \eta^{-} m_T^Q - \left(\beta_T + \frac{\eta^{+}}{n_T} q\right) m_T$$

$$\frac{dm_T^Q}{dt} = \frac{\eta^{+}}{n_T} q\ m_T - \left(\beta_T^Q + \eta^{-}\right) m_T^Q$$

$$\frac{dq}{dt} = n_T \alpha_Q + \left(\beta_T^Q + \eta^{-}\right) m_T^Q - \left(\beta_Q + \frac{\eta^{+}}{n_T}\ m_T\right) q$$

$$\frac{dm_C}{dt} = n_C \alpha_C - \beta_C\ m_C$$

$$\frac{dp_T}{dt} = \gamma_T^{Eff} - \delta_T\ p_T$$

$$\frac{dp_C}{dt} = \gamma_C^{Eff} - \delta_C\ p_C$$

The numerical values for the rescaling factors n_T and n_C are reported in **Supplementary Table 5**. To calculate their values, a general ribosome footprint size of ~30 nt (4, 5), which is equivalent to ~10 amino acids, is considered. Since the two genes have the same transcript length of ~1 kbp (see the model parameter values provided in **Supplementary Note 6**), then the rescaling factors can be calculated as:

$$n_T = n_C = \frac{1\ kbp}{30\ bp} \simeq\ 33$$

which corresponds to the maximum number of translating ribosomes allowed to simultaneously translate a single mRNA transcript.

Supplementary Note 4. Analytical steady-state solution of the resource-aware model.

The RNA dynamics can be approximated to a quasi-steady state in order to obtain an analytical steady-state solution of the model presented in **Supplementary Note 1**. Therefore, assuming that the RNA concentrations m_T, m_T^Q, q, and m_C reach their steady state faster than the protein concentrations p_T and p_C, we can make a QSSA for the RNA dynamics in the reduced model described in **Supplementary Note 1**. Such an assumption is biologically reasonable since RNA species are typically produced and degraded faster than protein species. Assuming a quasi-steady-state approximation of the RNA dynamics in the reduced ODE model (**Supplementary Note 1**), we obtain the following algebraic system of equations:

$$\frac{dm_T}{dt} \approx 0 \Rightarrow n_T \alpha_T + \eta^- \overline{m}_T^Q - \left(\beta_T + \frac{\eta^+}{n_T} \overline{q}\right) \overline{m}_T = 0$$

$$\frac{dm_T^Q}{dt} \approx 0 \Rightarrow \frac{\eta^+}{n_T} \overline{q}\, \overline{m}_T - \left(\beta_T^Q + \eta^-\right) \overline{m}_T^Q = 0$$

$$\frac{dq}{dt} \approx 0 \Rightarrow n_T \alpha_Q + \left(\beta_T^Q + \eta^-\right) \overline{m}_T^Q - \left(\beta_Q + \frac{\eta^+}{n_T} \overline{m}_T\right) \overline{q} = 0$$

$$\frac{dm_C}{dt} \approx 0 \Rightarrow n_C \alpha_C - \beta_C \overline{m}_C = 0$$

Solving this algebraic system of equations yields the following QSSA for the RNA dynamics:

$$\overline{m}_T = \frac{n_T \alpha_T}{\beta_T + \frac{\alpha_Q \eta^+ \beta_T^Q}{\beta_Q \left(\beta_T^Q + \eta^-\right)}}$$

$$\overline{m}_T^Q = \frac{n_T \alpha_T}{\beta_T^Q + \frac{\beta_T \beta_Q \left(\beta_T^Q + \eta^-\right)}{\alpha_Q \eta^+}}$$

$$\overline{q} = \frac{n_T \alpha_T}{\beta_Q}$$

$$\overline{m}_C = \frac{n_C \alpha_C}{\beta_C}$$

We then define the resource demand coefficients for the *miTarget* (ρ_T and ρ_T^Q) and *capacity monitor* (ρ_C) as:

$$\rho_T = \frac{\overline{m}_T}{\kappa_T} = \frac{n_T \alpha_T}{\kappa_T \left(\beta_T + \frac{\alpha_Q \eta^+ \beta_T^Q}{\beta_Q \left(\beta_T^Q + \eta^-\right)}\right)}$$

$$\rho_T^Q = \frac{\overline{m}_T^Q}{\kappa_T} = \frac{n_T\,\alpha_T}{\kappa_T\left(\beta_T^Q + \frac{\beta_T\,\beta_Q\,\left(\beta_T^Q+\eta^-\right)}{\alpha_Q\,\eta^+}\right)}$$

$$\rho_C = \frac{\overline{m}_C}{\kappa_C} = \frac{n_C\,\alpha_C}{\kappa_C\,\beta_C}$$

Hence, we can rewrite the effective translation rate constants (**Supplementary Note 1**) as:

$$\gamma_T^{Eff} = \gamma_T \cdot \frac{\rho_T}{1+\rho_C+\rho_T+\sigma\,\rho_T^Q} \cdot r^{Total}$$

$$\gamma_{T,Q}^{Eff} = \gamma_T \cdot \frac{\sigma\,\rho_T^Q}{1+\rho_C+\rho_T+\sigma\,\rho_T^Q} \cdot r^{Total}$$

$$\gamma_C^{Eff} = \gamma_C \cdot \frac{\rho_C}{1+\rho_T+\sigma\,\rho_T^Q+\rho_C} \cdot r^{Total}$$

Finally, we solve for the steady-state protein concentrations of both the *miTarget* and the *capacity monitor* by setting their ODE to zero:

$$\frac{dp_T}{dt} \approx 0 \Rightarrow \gamma_T^{Eff} + \gamma_{T,Q}^{Eff} - \delta_T\,\overline{p}_T = 0 \Rightarrow \overline{p}_T = \frac{\gamma_T^{Eff}+\gamma_{T,Q}^{Eff}}{\delta_T}$$

$$\frac{dp_C}{dt} \approx 0 \Rightarrow \gamma_C^{Eff} - \delta_C\,\overline{p}_C = 0 \Rightarrow \overline{p}_C = \frac{\gamma_C^{Eff}}{\delta_C}$$

Substituting the effective translation rate constants into the previous steady-state values, we obtain the following analytical steady-state solution for the protein concentrations:

$$\overline{p}_T = \frac{\gamma_T}{\delta_T} \cdot \frac{\rho_T+\sigma\,\rho_T^Q}{1+\rho_C+\rho_T+\sigma\,\rho_T^Q} \cdot r^{Total}$$

$$\overline{p}_C = \frac{\gamma_C}{\delta_C} \cdot \frac{\rho_C}{1+\rho_T+\sigma\,\rho_T^Q+\rho_C} \cdot r^{Total}$$

We note that the analytical steady-state solutions for the protein concentration of both genes depend on the three resource demand coefficients ρ_T, ρ_T^Q, and ρ_C.

Similarly, we can derive an analytical expression for the concentration of free ribosome at steady state:

$$\overline{r} = \frac{r^{Total}}{1+\rho_T+\sigma\,\rho_T^Q+\rho_C}$$

We note that the previous steady-state expression resembles a repressing Hill regulatory function that depends on the resource demand coefficients.

Finally, we can derive an analytical expression for the steady-state ribosomal densities for the *miTarget* (RD_T) and the *capacity monitor* (RD_C). Here, we define the ribosomal density as the number of translating ribosomes per transcript. Therefore, the ribosomal density for the *miTarget* gene at steady state can be calculated as:

$$RD_T = \frac{\bar{b}_T^{Tot}}{\bar{m}_T^{Tot} + \bar{b}_T^{Tot}} = \frac{\bar{b}_T + \bar{b}_T^{Q}}{\bar{m}_T + \bar{m}_T^{Q} + \bar{b}_T + \bar{b}_T^{Q}} = \frac{\frac{\rho_T + \sigma\,\rho_T^{Q}}{1+\rho_C+\rho_T+\sigma\,\rho_T^{Q}}\cdot r^{Total}}{\kappa_T\left(\rho_T + \sigma\,\rho_T^{Q}\right) + \frac{\rho_T + \sigma\,\rho_T^{Q}}{1+\rho_C+\rho_T+\sigma\,\rho_T^{Q}}\cdot r^{Total}} = \frac{\frac{r^{Total}}{1+\rho_T+\sigma\,\rho_T^{Q}+\rho_C}}{\kappa_T + \frac{r^{Total}}{1+\rho_T+\sigma\,\rho_T^{Q}+\rho_C}} = \frac{\bar{r}}{\kappa_T + \bar{r}}$$

Similarly the steady-state ribosomal density for the *capacity monitor* gene is given as:

$$RD_C = \frac{\bar{b}_C}{\bar{m}_C + \bar{b}_C} = \frac{\frac{\rho_C}{1+\rho_T+\sigma\,\rho_T^{Q}+\rho_C}\cdot r^{Total}}{\kappa_T\,\rho_C + \frac{\rho_C}{1+\rho_T+\sigma\,\rho_T^{Q}+\rho_C}\cdot r^{Total}} = \frac{\frac{r^{Total}}{1+\rho_T+\sigma\,\rho_T^{Q}+\rho_C}}{\kappa_C + \frac{r^{Total}}{1+\rho_T+\sigma\,\rho_T^{Q}+\rho_C}} = \frac{\bar{r}}{\kappa_C + \bar{r}}$$

Looking at these analytical expressions, we observe that the ribosomal densities depend on the steady-state concentration of free ribosomes via an activating Hill function. Although the analytical expressions for RD_T and RD_C look similar, they differ in terms of the half-saturation constants (κ_T and κ_C, respectively). These half-saturation constants correspond to the effective dissociation constant between the ribosome and the respective transcripts.

Supplementary Note 5. Resource-aware model to study reallocation of RNA degradation resources.

Here we describe the resource-aware deterministic model used to study the reallocation of RNA degradation resources (i.e., RNases) in **Fig. 4**. The deterministic model extends the ODE model described in **Supplementary Note 1** and considers both protein translation and RNA degradation. Once again, our model replaces reaction rates that involve shared cellular resources (in this case ribosomes and RNases) with effective reaction rates that account for the availability of these resources according to the overall demand from competing genes as shown in **Fig. 1b**. The key assumptions underlying the formulation of the model equations are as stated for the model in **Supplementary Note 1** except for *Assumption 2*, as we here assume that the shared cellular resource pool for RNA degradation is finite and constant. Briefly, we recap the assumptions in the following list:

1. The shared cellular resource pool for translation (ribosomes) is considered finite and constant.
2. The shared cellular resource pool for RNA degradation (RNases) is considered finite and constant.
3. Shared transcriptional resource pools (e.g., RNA polymerases) are not explicitly considered in the model. Such an assumption does not alter the findings of our study as shown in **Supplementary Note 2**.

To derive the deterministic model, we apply the law of mass action to the biochemical reactions shown in **Supplementary Fig. 2**. Here, we omit all biochemical reactions associated with mRNA translation (**Supplementary Fig. 1**). This is motivated by the fact that we use this model to study mRNA degradation and not protein production. Therefore, as demonstrated in **Supplementary Note 1**, the ODEs describing the RNA dynamics do not embed any term related to RNA translation if the QSSA of translating ribosomal complexes holds true. Hence, we obtain the following set of ODE:

$$\frac{dm_T}{dt} = \alpha_T + \eta^- m_T^Q + \beta_T^- s_T - \left(\beta_T^+ g + \eta^+ q + \mu\right) m_T$$

$$\frac{ds_T}{dt} = \beta_T^+ m_T g - \left(\beta_T^- + \beta_T^{Deg} + \mu\right) s_T$$

$$\frac{dm_T^Q}{dt} = \eta^+ q\, m_T + \beta_{T,Q}^- s_T^Q - \left(\beta_{T,Q}^+ g + \eta^- + \mu\right) m_T^Q$$

$$\frac{ds_T^Q}{dt} = \beta_{T,Q}^+ m_T^Q g - \left(\beta_{T,Q}^- + \beta_{T,Q}^{Deg} + \mu\right) s_T^Q$$

$$\frac{dq}{dt} = \alpha_Q + \beta_{T,Q}^{Deg} d_T^Q + \eta^- m_T^Q - \left(\beta_Q + \eta^+ m_T\right) q$$

$$\frac{dm_C}{dt} = \alpha_C + \beta_C^- s_C - \left(\beta_C^+ g + \mu\right) m_C$$

$$\frac{ds_C}{dt} = \beta_C^+ \, m_C \, g - \left(\beta_C^- + \beta_C^{Deg} + \mu\right) s_C$$

In contrast to the model described in **Supplementary Note 1**, the location of the miR-TS at the UTRs does not change the model behaviour (i.e., no boolean parameter σ is used here). All molecular species modelled here – including their description – are summarised in **Supplementary Table 6**, whilst all the model parameters – including the numerical values used to simulate the model (see **Supplementary Note 6** for the model parameterisation) – are listed in **Supplementary Table 7**.
In contrast to the translating ribosomal complexes described in **Supplementary Note 1**, the degrading *mRNA:RNase* complexes s_T, s_C, and s_T^Q do not reach their steady-state values faster than the other molecular species involved in the model. Hence, we cannot make a QSSA for the dynamics of the degrading *mRNA:RNase* complexes.
Assuming a limited amount of available degradation resources (g^{Total}), we can apply a resource conservation law on the total available amount of degradation resources:

$$g^{Total} = g + s_T + s_T^Q + s_C$$

which yields the concentration of free RNases:

$$g = g^{Total} - s_T + s_T^Q + s_C$$

Substituting the concentration of free RNases in the previous ODEs yields the resource-aware ODE model (**Fig. 4a**):

$$\frac{dm_T}{dt} = \alpha_T + \eta^- \, m_T^Q + \beta_T^- \, s_T - \left(\beta_T^{Eff} + \eta^+ \, q\right) m_T$$

$$\frac{ds_T}{dt} = \beta_T^+ \, m_T \, g - \left(\beta_T^- + \beta_T^{Deg} + \mu\right) s_T$$

$$\frac{dm_T^Q}{dt} = \eta^+ \, q \, m_T + \beta_{T,Q}^- \, s_T^Q - \left(\beta_{T,Q}^{Eff} + \eta^-\right) m_T^Q$$

$$\frac{ds_T^Q}{dt} = \beta_{T,Q}^+ \, m_T^Q \, g - \left(\beta_{T,Q}^- + \beta_{T,Q}^{Deg} + \mu\right) s_T^Q$$

$$\frac{dq}{dt} = \alpha_Q + \beta_{T,Q}^{Deg} \, d_T^Q + \eta^- \, m_T^Q - \left(\beta_Q + \eta^+ \, m_T\right) q$$

$$\frac{dm_C}{dt} = \alpha_C + \beta_C^- \, s_C - \beta_C^{Eff} \, m_C$$

$$\frac{ds_C}{dt} = \beta_C^+ \, m_C \, g - \left(\beta_C^- + \beta_C^{Deg} + \mu\right) s_C$$

where the effective degradation rate constants are defined as:

$$\beta_T^{Eff} = \beta_T^{+} g + \mu = \beta_T^{+} \left(g^{Total} - s_T + s_T^{Q} + s_C\right) + \mu$$

$$\beta_{T,Q}^{Eff} = \beta_{T,Q}^{+} g + \mu = \beta_{T,Q}^{+} \left(g^{Total} - s_T + s_T^{Q} + s_C\right) + \mu$$

$$\beta_C^{Eff} = \beta_C^{+} g + \mu = \beta_C^{+} \left(g^{Total} - s_T + s_T^{Q} + s_C\right) + \mu$$

These lumped parameters represent the core of the resource-aware model to study RNA degradation and reflect the dependency of the mRNA degradation rates on the effective availability of RNA degradation resources in the cell. Note that these lumped parameters do not depend on the location of the miR-TS at the UTRs. This explains why the location of the target sites at the UTRs does not alter the mRNA degradation profiles of either the *miTarget* or the *capacity monitor* genes.
The parameters used to simulate the model are summarised in **Supplementary Table 7**.

Supplementary Note 6. Model parameterisation.

To qualitatively simulate the different instances of the ODE model described in **Supplementary Notes 1-5**, we select a set of characteristic values so that the simulated dynamical behaviours are coherent with the experimental design of the two-gene circuit used in this study. More specifically, the two genes encode a DNA sequence to produce two different fluorescent proteins:

1. An mKate protein (red fluorescent protein) which represents the *miTarget* – or the miRNA sensor – in the circuit illustrated in **Fig. 1**.
2. An EGFP (enhanced green fluorescent protein) which represents the *Capacity monitor* in the circuit illustrated in **Fig. 1**.

The model parameters used to simulate the model in **Fig. 2** and **Fig. 3** of the main text are listed in **Supplementary Table 5**. To derive characteristic values for the transcription rate constants associated with these two exogenous genes, we first need to understand what could be the transcription rate constant associated with an endogenous gene. We consider an mRNA transcription rate of ~2.4 kbp/min (BNID: 111156, https://bionumbers.hms.harvard.edu/), which is equivalent to ~144 kbp/h, and an endogenous transcript length of ~1300 bp (CCNA2 gene; Gene ID: 890, https://www.ncbi.nlm.nih.gov/gene/890). Hence, the endogenous gene is transcribed at a rate of ~110 transcripts per hour. We assume an endogenous DNA concentration for the endogenous DNA sequence of ~0.001 nM (BNID: 108456, https://bionumbers.hms.harvard.edu/), hence the endogenous transcription rate constant is equivalent to ~0.1 nM/h. Keeping the transcription rate constant for the endogenous gene, we can then proceed to calculate the transcription rate constants for the exogenous genes. We assume that the transcription rate constants associated with the two exogenous genes differ from the one associated with the endogenous gene by an order of magnitude, therefore we set their values to ~1 nM/h. To set the transcription rate constant for the *miRNA*, we assume that the *miRNA* is transcribed at a rate of ~55 transcripts per hour, which is half of the value associated with the endogenous gene. This value is motivated by the fact that an endogenous miRNA may be encoded in an intron, hence the DNA sequence that needs to be transcribed can be longer than the miRNA itself. Also, a transcribed miRNA undergoes a maturation process that can further delay the miRNA biogenesis. Assuming an endogenous DNA concentration for the endogenous DNA sequence carrying the *miRNA* of ~0.001 nM (BNID: 108456, https://bionumbers.hms.harvard.edu/), the transcription rate constant for the *miRNA* is equivalent to ~0.05 nM/h. To set the translation rate constants, we consider a ribosome translocation rate of ~3.5 codons/s (6), which is equivalent to 12,600 codons/h, and a length of 238 amino acids for the *capacity monitor* (EGFP) and the *miTarget* (mKate). To set the RNA degradation rate constants, we consider a half-life for the *miTarget* and *capacity monitor* of ~3 hours (7), and a half-life for the *miRNA* of ~10 hours (8). The *miTarget:miRNA* mRNA degradation rate constant β_T^Q is assumed to be five-fold ($\lambda_T^Q \simeq 5$) larger than the *miTarget* mRNA degradation rate constant β_T. This characteristic value is inferred from the distribution of the parameter λ_T^Q obtained by repeating the model fitting 5,000 times (see **Supplementary Fig. 3**) and shown in **Supplementary Fig. 6** (mean value: λ_T^Q = 5.325). To set the protein degradation rate constants, we consider a half-life for the *miTarget* and *capacity monitor* of ~26 hours (9). RNA and protein degradation rate constants incorporate the cell

dilution rate into their values. The miRNA binding constant η^{+} is considered as an independent variable and thus is not set to a fixed value but rather spans a range of reasonable characteristic values. To set the association constants between a transcript and a ribosome, we simulate the ODE model with different values for the association constants κ_{T}^{+} and κ_{C}^{+} to understand how the miRNA-driven regulation changes the protein expression levels for the *miTarget* and the *capacity monitor* (**Supplementary Fig. 7**). To simplify the model, we assume that the two association constants are set to the same value, that is $\kappa_{T}^{+} = \kappa_{C}^{+}$. This is motivated by the fact that the two transcripts have similar RNA sequences, hence the binding rate constants κ_{T}^{+} and κ_{C}^{+} may only slightly differ from each other. Numerical simulations show that the qualitative trend of the protein expression levels does not change when varying κ_{T}^{+} and κ_{C}^{+}, but the absolute protein levels do as shown in **Supplementary Fig. 7**. Nevertheless, as we are interested in studying the qualitative trend for the protein expression levels upon miRNA action, we assume that the association constants κ_{T}^{+} and κ_{C}^{+} are set to a characteristic value that guarantees coherent protein expression levels comparable to those observed experimentally in **Fig. 2b**. To simplify the model, we assume that all the dissociation constants associated with the unbinding reactions (η^{-}, κ_{T}^{-}, and κ_{C}^{-}) are set to zero. We argue that such a choice does not affect the predictive power of the model since these dissociation constants are combined with other parameters throughout the model – more specifically, β_{T}^{Q} (i.e., $\beta_{T}^{Q} + \eta^{-}$), γ_{T} (i.e., $\gamma_{T} + \kappa_{T}^{-}$), and γ_{C} (i.e., $\gamma_{C} + \kappa_{C}^{-}$); respectively. Intuitively, the unbinding reactions cannot be predominant over the mRNA degradation (i.e., $\beta_{T}^{Q} + \eta^{-} \gg \eta^{-}$) and translation processes (i.e., $\gamma_{T} + \kappa_{T}^{-} \gg \kappa_{T}^{-}$, and $\gamma_{C} + \kappa_{C}^{-} \gg \kappa_{C}^{-}$), so their characteristic values can be embedded (and so neglected) into the mRNA degradation and translation rate constants without loss of generality. The total amount of translational resources available in the model is set to a characteristic value of 1,000 nM as reported in a previous study (10).
The model parameters used to simulate the model in **Fig. 4** are listed in **Supplementary Table 7**. Unless otherwise specified, all the model parameters have the same characteristic values as given for the model instance simulated in **Fig. 2** and **Fig.3**. To set the cell growth rate constant (equivalent to the dilution rate constant), we consider a cell division rate of ~24 hours (BNID: 106813, https://bionumbers.hms.harvard.edu/). The endogenous mRNA transcription rate is set to 0.1 nM/h, which accounts for the smaller concentration of an endogenous gene (i.e., ~0.001 nM; BNID: 108456, https://bionumbers.hms.harvard.edu/). To set the parameters associated with the mRNA degradation dynamics (β_{T}^{+}, β_{T}^{Deg}, $\beta_{T,Q}^{+}$, $\beta_{T,Q}^{Deg}$, β_{E}^{+}, β_{E}^{Deg}, and g^{Total}), we conduct an empirical search on the model parameters so as to obtain a simulated dynamical behaviour coherent with the mRNA degradation dynamics of an endogenous gene similar to the ones considered in this study (CCNA2 and eIF4E). Similarly to the previous case, we assume that all the dissociation constants associated with the unbinding reactions (η^{-}, β_{T}^{-}, and β_{E}^{-}) are set to zero. The miRNA binding constant η^{+} is still considered as an independent variable, which takes values from a discrete set of reasonable values aimed to study the reallocation of RNA degrading resources.

References

1. Cella,F., Wroblewska,L., Weiss,R. and Siciliano,V. (2018) Engineering protein-protein devices for multilayered regulation of mRNA translation using orthogonal proteases in mammalian cells. *Nature Communications*, **9**.

2. Frei,T., Cella,F., Tedeschi,F., Gutiérrez,J., Stan,G.-B., Khammash,M. and Siciliano,V. (2020) Characterization and mitigation of gene expression burden in mammalian cells. *Nat. Commun.*, **11**, 4641.

3. Qian,Y., Huang,H.-H., Jiménez,J.I. and Del Vecchio,D. (2017) Resource Competition Shapes the Response of Genetic Circuits. *ACS Synth. Biol.*, **6**, 1263–1272.

4. Ingolia,N.T., Ghaemmaghami,S., Newman,J.R.S. and Weissman,J.S. (2009) Genome-wide analysis in vivo of translation with nucleotide resolution using ribosome profiling. *Science*, **324**, 218–223.

5. Gobet,C. and Naef,F. (2017) Ribosome profiling and dynamic regulation of translation in mammals. *Curr. Opin. Genet. Dev.*, **43**, 120–127.

6. Yan,X., Hoek,T.A., Vale,R.D. and Tanenbaum,M.E. (2016) Dynamics of Translation of Single mRNA Molecules In Vivo. *Cell*, **165**, 976–989.

7. He,L., Binari,R., Huang,J., Falo-Sanjuan,J. and Perrimon,N. (2019) In vivo study of gene expression with an enhanced dual-color fluorescent transcriptional timer. *Elife*, **8**.

8. Zlotorynski,E. (2019) Insights into the kinetics of microRNA biogenesis and turnover. *Nat. Rev. Mol. Cell Biol.*, **20**, 511.

9. Nash,K.L. and Lever,A.M.L. (2004) Green fluorescent protein: green cells do not always indicate gene expression. *Gene Ther.*, **11**, 882–883.

10. Jones,R.D., Qian,Y., Siciliano,V., DiAndreth,B., Huh,J., Weiss,R. and Del Vecchio,D. (2020) An endoribonuclease-based feedforward controller for decoupling resource-limited genetic modules in mammalian cells. *Nat. Commun.*, **11**, 5690.
